# Mathematical model for glutathione dynamics in the retina

**DOI:** 10.1038/s41598-023-37938-9

**Published:** 2023-07-07

**Authors:** Atanaska Dobreva, Erika Tatiana Camacho, María Miranda

**Affiliations:** 1 Department of Mathematics, Augusta University, Augusta, GA 30912 USA; 2 University of Texas at San Antonio, San Antonio, TX 78249 USA; 3 School of Mathematical and Statistical Sciences, Arizona State University, Tempe, AZ 85281 USA; 4 Department of Biomedical Sciences, Faculty of Health Sciences, Institute of Biomedical Sciences, Cardenal Herrera-CEU University, CEU Universities, 46115 Valencia, Spain

**Keywords:** Computational models, Differential equations, Numerical simulations

## Abstract

The retina is highly susceptible to the generation of toxic reactive oxygen species (ROS) that disrupt the normal operations of retinal cells. The glutathione (GSH) antioxidant system plays an important role in mitigating ROS. To perform its protective functions, GSH depends on nicotinamide adenine dinucleotide phosphate (NADPH) produced through the pentose phosphate pathway. This work develops the first mathematical model for the GSH antioxidant system in the outer retina, capturing the most essential components for formation of ROS, GSH production, its oxidation in detoxifying ROS, and subsequent reduction by NADPH. We calibrate and validate the model using experimental measurements, at different postnatal days up to PN28, from control mice and from the *rd1* mouse model for the disease retinitis pigmentosa (RP). Global sensitivity analysis is then applied to examine the model behavior and identify the pathways with the greatest impact in control compared to RP conditions. The findings underscore the importance of GSH and NADPH production in dealing with oxidative stress during retinal development, especially after peak rod degeneration occurs in RP, leading to increased oxygen tension. This suggests that stimulation of GSH and NADPH synthesis could be a potential intervention strategy in degenerative mouse retinas with RP.

## Introduction

Reactive oxygen species (ROS) are byproducts of the normal metabolic function of any cell in the body and damage proteins, lipids and nucleotides, thereby interfering with a myriad of important cellular processes^1^. The retina of the eye constitutes an ideal model to study ROS and cellular redox/antioxidant defense systems. Due to light absorption, exposure to oxygen tension, and high metabolic and energy demands, the retina is an environment highly susceptible to the formation of ROS, including oxidants and free radicals, which damage cellular proteins and lipid membranes^1–4^. The retina houses the sensory cells responsible for vision, rod and cone photoreceptors, which are the most metabolically active cells in the body^2,5^. Among the systems of retinal cells to mitigate ROS and manage oxidative stress, a principal one is glutathione (GSH), and it relies on nicotinamide adenine dinucleotide phosphate (NADPH)^1^. The interplay between ROS and the GSH redox system underlies the vitality of many other cells in the body, as well.

The central nervous system (CNS) depends almost exclusively on glucose to obtain energy, and the retina is part of the CNS. The retina has the highest relative energy consumption compared to other tissues, and its energy metabolism relies heavily on a regular supply of glucose from the bloodstream^6^. Glucose from the choroidal circulation must pass through the retinal pigment epithelium (RPE) in order to reach the photoreceptors^7^. In vertebrates, photoreceptors are some of the cells with most active metabolism, and even compared to the brain, they use more glucose as a metabolic fuel^8^. CNS brain neurons are mainly fueled by glucose metabolized through OXPHOS; however, in photoreceptors most glucose is metabolized into lactate by aerobic glycolysis^9^. The lactate is then exported to the RPE and Müller glial cells for their nourishment^7^. While glucose is the preferred substrate for energy production in retinal neurons, under certain circumstances, including cell degeneration and glucose-deprived conditions, they use alternate fuel substrates in order to improve metabolic efficiency^9–11^. For example, glucose is the main substrate that photoreceptors utilize to create ATP in vivo^12^, but they can oxidize lipids, lactate, and use amino acids, fatty acids (such as palmitate) and other intermediate substrates to produce ATP, especially under glucose-deprived conditions^10–12^.

As part of metabolic processes in photoreceptors, glucose is converted to pyruvate. A portion of pyruvate enters the mitochondria where it is used to fuel energy production via the citric acid cycle and oxidative phosphorylation (OXPHOS). OXPHOS requires the presence of oxygen, and oxygen conversion into water occurs in the inner mitochondrial membrane. If the conversion process is interrupted and cannot be completed, there is leakage of electrons from the mitochondrial respiratory chain^2,13^, and this increases the generation of superoxide anions ($\text{ O}^{-}_{2}$)^14^, which are precursors of most types of ROS^13^. This occurs in RPE cells and other retinal cells as well.

In addition to $\text{ O}^{-}_{2}$, other types of ROS include hydroxyl radical ($\text{ OH}^{\bullet }$), hydrogen peroxide ($\text{ H}_{2}\text{ O}_{2}$), and singlet oxygen (${^1}{\text{ O }}{_2}$)^15^. When oxygen accepts one electron, it becomes $\text{ O}^{-}_{2}$, and if it subsequently takes another electron and two protons, it becomes H$_2$O$_2$, and an additional electron splits H$_2$O$_2$, into $\text{ OH}^{\bullet }$ and a hydroxyl anion ($\text{ OH}^{-}$) via Fenton reaction^16^. Apart from ROS being created as a result of oxidative glucose metabolism, the constant exposure of retinal cells to sunlight and artificial light triggers the production of ROS^16,17^, especially with photosensitizers present, such as retinoids^16,18^, and leads to photo-oxidation^19^. Though the cornea and lens absorb most of the ultraviolet (UV) radiation, a small fraction of it can reach the retina. In this sense, ROS can arise as a result of photochemical reactions and exposure to UV light. Blue light, the high-energy short-wave light, can penetrate the cornea and lens, reaching the retina and ultimately inducing photochemical damage^16,20^. Photo-oxidative damage occurs when light interacts with endogeneous chromophore molecules, such as visual pigments. The absorption of light by chromophores puts them in a state of high excitation marked by a tendency to undergo rapid chemical reactions with other molecules, including molecular oxygen, which can lead to the production of ROS^3,4,21^. Photo-oxidation is exacerbated during retinal degeneration and progressive loss of photoreceptors, as the choroid cannot alter the inflow of oxygen supply in response to the changing retinal environment, so the remaining photoreceptors become exposed to a higher oxygen level^22^.

Formation of ROS via photo-oxidation affects the rods more severely since these cells detect dim light to provide night vision and are more vulnerable to damage induced by daylight^23^. ROS are also created as a result of phagocytosis by the RPE of the photoreceptors’ light absorbing photo-pigments contained in the outer segments (OS)^24^. The OS membranes are composed of lipids with high concentration of polyunsaturated fatty acids (PUFAs), which are prone to being oxidized by ROS^1^. Both rods and cones shed approximately 10% of their OS daily, and the discarded OS tips are ingested by the RPE^25^. Inefficiency of the phagocytosis process leads to the formation of residual lipid-protein granules that cannot be degraded, known as lipofuscin, and generation of ROS occurs from the constant exposure of lipofuscin to light and oxygen tension^1^.

The retina has enzymatic mechanisms for scavenging of free radicals as well as intrinsic antioxidant and redox systems for dealing with other types of ROS^17^. Singlet oxygen is primarily detoxified by carotenoids (naturally occurring pigments in the macula) like lutein and zeaxanthin^1^. Superoxide dismutase is an enzyme converting superoxide anion to oxygen and hydrogen peroxide. The latter are less harmful components that do not have as much oxidation power^14^. Hydrogen peroxide is mainly neutralized by the enzyme catalase, which turns hydrogen peroxide into water and oxygen, and the tripeptide glutathione (GSH), which converts hydrogen peroxide into water through a reducing reaction catalyzed by the enzyme glutathione peroxidase (GPX). GSH is also capable of reducing other ROS through nonenzymatic reactions^1^, and these reactions result in significant degradation of GSH^26^.

In addition to ROS, GSH can be involved in the detoxification of fatty acid, phospholypid and cholesterol hydroperoxides as well as reactive aldehydes. In the course of reducing reactions GSH is converted to its oxidized form (GSSG). Glutathione reductase (GSR), which uses NADPH as a cofactor, converts GSSG back to GSH^1,27^. Under normal conditions, the ratio of GSH to GSSG ranges from 100:1 to 20:1. Under oxidative stress, the GSH/GSSG ratio ranges from 5:1 to 1:1^28^.

In photoreceptors, NADPH is produced mostly from glucose-6-phosphate (G6P) through the pentose phosphate pathway (PPP)^5^. Apart from being a necessary co-factor for the reductases of the cellular antioxidant systems, including GSR which reduces $\text{ GSSG }$, $\text{ NADPH }$ also participates in the synthesis of fatty acids and in the cones’ visual cycle. Fatty acids are constituents of phospholipids needed to create cell membranes and nucleotides^29,30^. $\text{ NADPH }$ serves as a co-factor, providing reducing equivalents, in particular hydrides (negatively charged hydrogen ions), for the biosynthetic enzymes keto reductase and enoyl reductase, which are involved in the anabolic reactions to create fatty acids needed to regenerate the lipid-rich photoreceptor OS. This process converts $\text{ NADPH }$ to its oxidized form $\text{ NADP}^{+}$^31^. Additionally, NADPH is used in the visual cycle of photoreceptors for the conversion of the isomerized light-absorbing chromophore retinal back to its native state^32^.

GSH biosynthesis involves two enzyme-catalyzing steps that require ATP. First, glutamate-cysteine ligase (GCL) catalyzes the conjugation of cysteine and glutamate, which results in the formation of $\gamma$-glutamylcysteine. Next, glycine is added to $\gamma$-glutamylcysteine to generate $\gamma$-glutamyl-cysteinyl-glycine or GSH, and this second step is catalyzed by glutathione synthase^33^. The presence of ROS stimulates the production of GSH by increasing cystine uptake and promoting the expression of GCL^34^. In addition, GSH regulates the balance of its production and utilization by feedback inhibition of the reaction catalyzed by GCL^33,35^.

Imbalances in the GSH system can contribute to the development of retinal diseases, such as age-related macular degeneration, glaucoma, diabetic retinopathy, and retinitis pigmentosa (RP). In RP genetic mutations lead to death of rod photoreceptors, and as a significant number of rods is lost, then cones begin to degenerate^2^. The *rd1* mouse is a common animal model used to study RP in which the expression of over 60 genes, related to proliferation, apoptosis, and transcription, is modified via a mutation in the phosphodiesterase-6 gene, which affects the metabolism of cyclic guanosine monophosphate. Due to these genetic alterations, the *rd1* retina suffers metabolic disturbances that through various mechanisms cause degeneration of rods^36^. The peak in rod degeneration in the *rd1* mouse occurs between postnatal day (PN) 11 and PN13, and by PN20 all rods have degenerated^37^.

**Figure 1:**
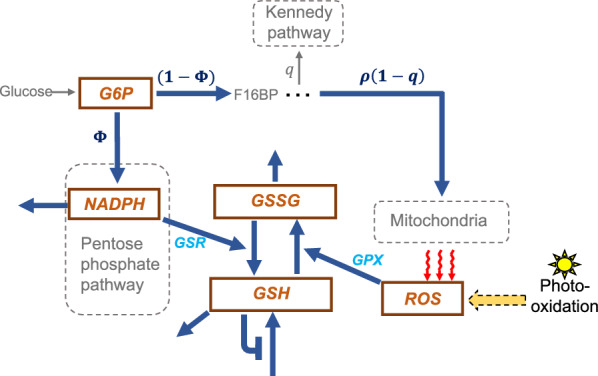
Diagram for model of the GSH system and its inputs. Model outputs are designated with brown boxes; processes and interactions are depicted with blue arrows. G6P is produced from glucose. A proportion $\Phi$ of G6P is diverted to the PPP to produce NADPH. A proportion $(1-\Phi )$ of G6P is used to synthesize F16BP, of which proportion *q* is diverted to the Kennedy pathway for the production of lipids, and proportion $(1-q)$ is directed to the pathway for pyruvate synthesis. $\rho$ approximates the proportion of pyruvate diverted to OXPHOS in the mitochondria. ROS is generated due to photo-oxidation and as a consequence of electron leakage from the mitochondrial respiratory chain. GSH regulates its own production via feedback inhibition. In the course of detoxifying ROS, GSH is oxidized to GSSG, and the conversion of GSSG back to GSH relies on NADPH. G6P: glucose-6-phosphate, F16BP: fructose-1,6-bisphosphate, PPP: pentose phosphate pathway, ROS: reactive oxygen species, GSH: reduced form of glutathione, GSSG: oxidized form of glutathione.

GSH redox dynamics have been included within kinetic models for metabolism of fungi^38^, yeast^39^, erythrocytes^40^, and liver^41–46^, kidney^47^, immune^48^ and cancer cells^49,50^. However, the GSH redox system has not been studied in the retina from a mathematical modeling perspective. To address this knowledge gap, here we develop a mathematical model that incorporates the most essential molecular elements for NADPH production, ROS generation, GSH synthesis, and detoxification of ROS by GSH in the outer retina, formed by photoreceptors and the RPE (see Fig. 1). Since visual function depends on normal outer retina operations, which are susceptible to disruption due to the highly oxidative retinal environment, it is crucial to understand the role of the GSH redox system in maintaining homeostasis. We explore the glucose divergence into the PPP and OXPHOS as well as the creation of ROS through mitochondrial leakage and the mitigation of ROS by the GSH redox system. Taking a mathematical modeling and analysis approach allows us to study the relative contribution of each pathway captured in the resulting model. We calibrate and validate the model with experimental data from control C3H mice and from the *rd1* mouse model for the disease RP^37^. Additionally, we perform global sensitivity analysis to examine which pathways have the greatest impact on ROS and the GSH redox system in control compared to RP conditions.

## Methods

### Mathematical model

The model diagram in Fig. 1 illustrates the essential reactions and feedback mechanisms among ROS, GSH, GSSG, NADPH and G6P in the outer retina, which we focus on and model as a system of ordinary differential equations (ODEs), where all parameters are non-negative. The concentrations of ROS, GSH, GSSG, NADPH and G6P are given by the model variables [ROS], [GSH], [GSSG], [NADPH] and [G6P], respectively.

NADPH plays an essential role in the gluthatoine redox system of the photoreceptors and the RPE. NADPH, derived from glucose via the PPP, is the ultimate electron donor that reduces the downstream proteins in the GSH antioxidant system. Thus, in addition to being a preferred energy substrate of retinal neurons, glucose is also a key driver of the redox system^16^. Consistent with this, glucose, defined as *G* in our model, is the substrate for the reaction rate in the production of [G6P] (Eq. (5) below), which is modeled as an allosteric reaction. As mentioned in the Introduction, there are many factors that contribute to ROS including oxygen and blue light that can penetrate the cornea and lens and induce photochemical damage^16,20^. However, in this study we are not explicitly modeling how ROS is created from photo-oxidation; our mathematical model incorporates in the term *r* all the factors that contribute to ROS and that are not part of the metabolism of glucose.

#### Reaction rates

Certain forms of ROS, such as hydrogen peroxide, serve as a substrate for the enzyme glutathione peroxidase (GPX), which catalyzes the oxidation of glutathione to GSSG. GSH is also a co-substrate used by GPX to reduce ROS^51^. Thus, GSH serves to reduce ROS, and both act as substrates in the detoxifying reaction. Assuming a sequential reaction, where the enzyme, GPX, must bind to both substrates, ROS and GSH, for the reaction producing GSSG to occur and letting ROS and GSH to be the leading and following substrates, respectively,

$$\begin{aligned} { \nu _{[\text{ GSSG}]}=\frac{V_{\text{ max}_{{oxid}}}[\text{ ROS}][\text{ GSH}]}{K_{{s}_{\left[ \text{ ROS }\right] }}K_{{m}_{\left[ \text{ GSH }\right] }}+K_{{m}_{\left[ \text{ ROS }\right] }}[\text{ GSH}]+K_{{m}_{\left[ \text{ GSH }\right] }}[\text{ ROS}]+[\text{ ROS}][\text{ GSH}]} } \end{aligned}$$

is the the reaction rate for GSSG.

Here, $V_{\text{ max }}$ is the maximal velocity of the reaction with both substrates, ROS and GSH, present at saturating concentrations, $K_{{m}}$ is the concentration of one substrate, say ROS in the case of $K_{{m}_{\left[ \text{ ROS }\right] }}$, necessary to achieve half $V_{\text{ max }}$ when the other substrate, GSH, is present at a saturating concentration, and $K_{{s}}$ is the dissociation constant from the enzyme, GPX^52,53^. This approach is also applied to the rate of the reaction reducing GSSG, facilitated by NADPH. Thus,

$$\begin{aligned} { \nu _{[\text{ GSH}]}=\frac{V_{\text{ max}_{{reduc}}}[\text{ GSSG}][\text{ NADPH}]}{K_{{s}_{\left[ \text{ GSSG }\right] }}K_{{m}_{\left[ \text{ NADPH }\right] }}+K_{{m}_{\left[ \text{ GSSG }\right] }}[\text{ NADPH}]+K_{{m}_{\left[ \text{ NADPH }\right] }}[\text{ GSSG}]+[\text{ GSSG}][\text{ NADPH}]} } \end{aligned}$$

where $\nu _{[\text{ GSH}]}$ describes the rate of the reaction in which $\text{ GSSG }$ is reduced by glutathione reductase (GSR) and returns to the form $\text{ GSH }$, which requires $\text{ NADPH }$ and GSSG as co-substrates. For further information on the form of the terms $v_{[GSSG]}$ and $v_{[GSH]}$, we refer the interested reader to Enzyme Kinetics: Catalysis & Control (2010) by Daniel L. Purich^53^.

#### Differential equations

We model the dynamics of the concentration of NADPH as follows

1 $$\begin{aligned} \frac{d[\text{ NADPH}]}{dt}=\frac{V_{n}\left[ \text{ G6P }\right] ^{2}}{K^{2}_{n}+\left[ \text{ G6P }\right] ^{2}}\Phi -\theta _{\left[ \text{ NADPH }\right] }\nu _{[\text{ GSH}]}-n_{\left[ \text{ NADPH }\right] }[\text{ NADPH}]. \end{aligned}$$

    NADPH is produced from G6P via the PPP (defined by $\frac{V_{n}\left[ \text{ G6P }\right] ^{2}}{K^{2}_{n}+\left[ \text{ G6P }\right] ^{2}}\Phi$, where $\Phi$ quantifies the proportion of G6P diverted to the PPP)^54^. NADPH is lost due to being involved in the conversion of $\text{ GSSG }$ to $\text{ GSH }$ (defined by $\theta _{\left[ \text{ NADPH }\right] }\nu _{[\text{ GSH}]}$)^5,54^ as well as from utilization in other biosynthetic processes (defined by $n_{\left[ \text{ NADPH }\right] }[\text{ NADPH}]$)^29–31^.

The temporal behavior of the concentration of ROS is described with the equation

2 $$\begin{aligned} \frac{d[\text{ ROS}]}{dt}=\rho (1-q)\frac{V_{f}\left[ \text{ G6P }\right] ^{2}}{K^{2}_{f}+\left[ \text{ G6P }\right] ^{2}}(1-\Phi )+r-\theta _{\left[ \text{ ROS }\right] }\nu _{[\text{ GSSG}]}-n_{\left[ \text{ ROS }\right] }[\text{ ROS}]. \end{aligned}$$

    ROS is created due to photo-oxidation and other factors that are not part of glucose metabolism (represented by *r*)^15,17^. ROS is also generated as a consequence of electron leakage from the mitochondrial respiratory chain, illustrated by $\rho (1-q)\frac{V_{f}\left[ \text{ G6P }\right] ^{2}}{K^{2}_{f}+\left[ \text{ G6P }\right] ^{2}}(1-\Phi )$, where $\frac{V_{f}\left[ \text{ G6P }\right] ^{2}}{K^{2}_{f}+\left[ \text{ G6P }\right] ^{2}}(1-\Phi )$ reflects the reaction of G6P down the glycolysis pathway and *q* is the proportion of the metabolite diverted to the Kennedy pathway for the production of lipids. The term $(1-q)\frac{V_{f}\left[ \text{ G6P }\right] ^{2}}{K^{2}_{f}+\left[ \text{ G6P }\right] ^{2}}(1-\Phi )$ approximates the production of pyruvate, and $\rho$ quantifies the proportion of pyruvate diverted to the mitochonria, where in the process of OXPHOS, electron leakage from the mitochondrial respiratory chain leads to the generation of ROS^5,15^. The level of ROS decreases due to enzymatic reduction by $\text{ GSH }$ (which we model with the term $\theta _{\left[ \text{ ROS }\right] }\nu _{[\text{ GSSG}]}$)^1,5,54^ as well as nonenzymatic reduction by $\text{ GSH }$ and activities of other antioxidant compounds (represented by $n_{\left[ \text{ ROS }\right] }[\text{ ROS}]$)^1,17^.

The dynamics of the concentration of GSH are modeled as

3 $$\begin{aligned} \frac{d[\text{ GSH}]}{dt}=\frac{s_{\left[ \text{ GSH }\right] }}{1+\sigma _{\left[ \text{ GSH }\right] }[\text{ GSH}]}+\nu _{[\text{ GSH}]}-\nu _{[\text{ GSSG}]}-n_{\left[ \text{ GSH }\right] }[\text{ GSH}]. \end{aligned}$$

    GSH inhibits its own production^1,33–35^, which in equation (3) is defined by the term $\frac{s_{\left[ \text{ GSH }\right] }}{1+\sigma _{\left[ \text{ GSH }\right] }[\text{ GSH}]}$, where $s_{\left[ \text{ GSH }\right] }$ represents the rate at which GSH is produced, and the parameter $\sigma _{\left[ \text{ GSH }\right] }$ controls the strength of inhibition that GSH exerts on its production. The GSH level increases due to the conversion of GSSG to GSH (characterized by $\nu _{[\text{ GSH}]}$) and decreases as GSH is converted to GSSG due to enzymatic detoxification of ROS (defined by the reaction rate $\nu _{[\text{ GSSG}]}$)^1^. In addition, GSH is lost from utilization in nonenzymatic ROS reduction and detoxification of other harmful compounds as well as degradation (and we incorporate this loss by $n_{\left[ \text{ GSH }\right] }[\text{ GSH}]$)^1,26^. As GSH cannot readily cross the cellular membrane due to its biochemical properties^35^, we assume that GSH excretion is negligible and do not account for it in the equation.

The dynamics of the concentration of GSSG are governed by the equation

4 $$\begin{aligned} \frac{d[\text{ GSSG}]}{dt}=\nu _{[\text{ GSSG}]}-\nu _{[\text{ GSH}]}-n_{\left[ \text{ GSSG }\right] }[\text{ GSSG}]. \end{aligned}$$

    The GSSG concentration increases as GSH is oxidized in enzymatic detoxification of ROS (defined by $\nu _{[\text{ GSSG}]}$). The pool of GSSG is depleted as GSSG is reduced back to GSH (represented by $\nu _{[\text{ GSH}]}$)^1^ and due to the cellular excretion of GSSG (defined as $n_{\left[ \text{ GSSG }\right] }[\text{ GSSG}]$)^51^. In the extracellular space GSSG is broken down into glutamate, glycine and cysteine residues^51^.

Finally, the dynamics of G6P are described as follows

5 $$\begin{aligned} \frac{d[\text{ G6P}]}{dt}=\frac{V_{p} G^{2}}{K^{2}_{p}+G^{2}}-\frac{V_{f}\left[ \text{ G6P }\right] ^{2}}{K^{2}_{f}+\left[ \text{ G6P }\right] ^{2}}(1-\Phi )-\frac{V_{n}\left[ \text{ G6P }\right] ^{2}}{K^{2}_{n}+\left[ \text{ G6P }\right] ^{2}}\Phi , \end{aligned}$$

where $\frac{V_{p} G^{2}}{K^{2}_{p}+G^{2}}$ quantifies the reaction rate of glucose conversion to G6P, and the second and third term represent the diversions of G6P to glycolysis and the PPP, respectively. The input *G* represents the concentration of glucose in the retina.

### Model analysis

#### Selection of parameters for estimation

The methodology to select parameters for estimation involves first conducting local sensitivity analysis (LSA) to obtain a set of important parameters, and then determining from this set of parameters which are identifiable, that is for which parameters a value can be identified from the data, taking into account the model structure. LSA shows the level of impact small changes in the parameters/inputs of the model have on the model’s dependent variables/outputs, if the parameters are varied one at a time. In LSA, a parameter is classified as important if small changes in the parameter have a large impact on the output level. The impact of a parameter on the output is indicated by the magnitude of the local sensitivity coefficient $\frac{\partial y}{\partial \xi }$, where *y* is the output and $\xi$ is the parameter. The local sensitivity coefficients are calculated with the Direct Method, where a system of sensitivity differential equations is solved directly together with the model differential equations, $\frac{dy}{dt}=f(y,\xi )$, using a numerical ODE solver.

According to the theory of local sensitivity analysis of ODE systems, where the system variables, *y*, are smooth differentiable functions, to obtain the sensitivity differential equations, both sides of $\frac{dy}{dt}=f(y,\xi )$ are differentiated with respect to the parameters, $\xi$, that is $\frac{\partial }{\partial \xi }\left( \frac{dy}{dt}\right) =\frac{\partial }{\partial \xi }\left( f(y,\xi )\right)$^55,56^. The chain rule for differentiation is applied on the right-hand side, $\frac{\partial }{\partial \xi }\left( f(y,\xi )\right)$, yielding $\frac{\partial f}{\partial y}\frac{\partial y}{\partial \xi }+\frac{\partial f}{\partial \xi }\frac{\partial \xi }{\partial \xi }$. The calculus rule for interchanging the order of differentiation in a mixed derivative is applied to the left-hand side to interchange the order of differentiation of *y* and obtain $\frac{\partial }{\partial \xi }\left( \frac{dy}{dt}\right)$ equal to $\frac{d}{dt}\left( \frac{\partial y}{\partial \xi } \right)$^55,56^.

Thus, the sensitivity differential equations are of the form

6 $$\begin{aligned} \frac{d}{dt}\left( \frac{\partial y}{\partial \xi }\right) = \frac{\partial f}{\partial y}\frac{\partial y}{\partial \xi }+\frac{\partial f}{\partial \xi }\frac{\partial \xi }{\partial \xi }= \frac{\partial f}{\partial y}\frac{\partial y}{\partial \xi }+\frac{\partial f}{\partial \xi }, \end{aligned}$$

where the derivative of the model equations’ right-hand side with respect to the system variables is $\frac{\partial f}{\partial y}$ and with respect to the parameters is $\frac{\partial f}{\partial \xi }$. The initial condition for each sensitivity differential equation is zero^57^. The initial conditions for the model equations, Eq. (1)-(5), are as specified in Table 3.

**Figure 2:**
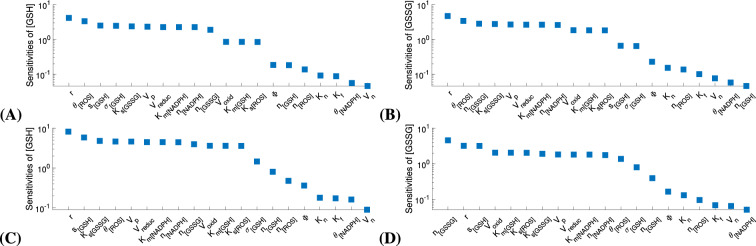
LSA results for [GSH] and [GSSG] for control (**A**, **B**) and RP case (**C**, **D**), where the most influential parameters have sensitivity coefficient greater than a threshold of $10^{-1}$. For ease of readability, $V_{\text{ max}_{{oxid}}}$ is displayed as $V_{\text{ oxid }}$ and $V_{\text{ max}_{{reduc}}}$ is displayed as $V_{\text{ reduc }}$.

Parameters with normalized local sensitivity coefficient $\frac{\partial y}{\partial \xi } \frac{\xi }{y}$ greater than a specified threshold are regarded as influencial and considered for estimation. In general, the threshold is a value greater than $10\sqrt{tol}$, where *tol* is the integration tolerance in solving the model numerically (for this study $tol=10^{-8}$). Note that the solutions of the sensitivity differential equations are vectors of values over the time interval we consider (PN11 through PN28, expressed in minutes), so in calculating the normalized local sensitivity coefficients (shown in Fig. 2), we used the Euclidean norm of the vectors of local sensitivity coefficients.

In order to determine a set of identifiable parameters from the influencial parameters found with LSA we used structured correlation analysis, in which pairwise correlation coefficients are computed from the entries in the covariance matrix *C* for influential model parameters, obtained by $C=(L^{T}L)^{-1}$, where *L* is the local sensitivity matrix containing the coefficients of important parameters to which the model outputs are sensitive to^58^. The pairwise correlation coefficients are computed as follows

7 $$\begin{aligned} cor_{ij} = \frac{C_{ij}}{\sqrt{C_{ii}C_{jj}}}. \end{aligned}$$

    If the magnitudes of the pairwise correlation coefficients are less than 0.90, the parameters in the pair are considered identifiable and can be estimated together^58^.On the other hand, parameters with pairwise coefficient greater than 0.90 in magnitude are considered correlated and thus cannot be estimated together.

#### Model calibration and validation

For calibration and validation of the mathematical model we used data from experiments with *rd1* and control C3H mice where the retinal concentrations of GSH and GSSG were measured at different postnatal days (PN). Measurements at PN11, PN17, PN28 were available from a previously published study (Gimeno-Hernández et al. (2020))^37^. The protocol of the experimental study was approved by the Animal Ethics Committee of CEU Cardinal Herrera University and in accordance with the Association for Research in Vision and Ophthalmology’s (ARVO) guidelines for use of animals in eye research. The experimental procedures involved euthanization of mice followed by dissecting and homogenating the retinas from both eyes of each mouse and utilizing the Reed method^59^ to measure the concentrations of GSH and GSSG, and the Lowry method^60^ to quantify protein concentrations^37^. We also used an unpublished data set with measurements for GSH and GSSG collected at PN13 and PN15 for control mice, and at PN12, PN13 and PN15 for *rd1* mice. The unpublished data was generated after the study of Gimeno-Hernández et al. (2020), but before the conception of this computational study. The mice were obtained from the Jackson Laboratory colony (The Jackson Labs, Bar Harbor, ME, USA) and housed in the facilities of the Research Unit of CEU Cardenal Herrera University (Valencia, Spain). The control and care of the mice were authorized by the CEU Cardenal Herrera University Committee for Animal Experiments, and the ARVO Statement for the Use of Animals in Ophthalmic and Vision Research as well as the ARRIVE guidelines were followed. Measurement of GSH, GSSG and protein concentration was done following the same experimental protocol as described in Gimeno-Hernández et al. (2020)^37^. All data are included in Table S2 in the Supplementary Information together with an explanation on conversion of data values to the units of the mathematical model.

To calibrate and validate the model, we split the available experimental data into a calibration set and a validation set. Calibration is done performing parameter estimation, and validation involves calculating goodness of fit ($R^2$). Since at least three points are needed to calculate goodness of fit, the six data points from *rd1* mice we divided by including three points in the calibration set and three points in the validation set, with the first point always being in the calibration set. For the remaining data points, we explored grouping cases to account for the different possibilities of the points being included in either set. We also explored grouping cases in forming the calibration and validation sets with the five data points from control mice, where the need for three data points in the validation set necessitated repeating one of the calibration points in the validation set. The calibration and validation results for all grouping cases are presented in the Supplementary Information, and for both control and *rd1*, the case with best outcome, that is high goodness of fit value for both [GSH] and [GSSG], is included in the “Results” section.

The calibration algorithm conducts a search for values for the estimated parameters in order to find values such that the error, i.e., the difference between the calibration data measurements and the model output predictions is minimized. We employ the least squares method which minimizes the error function

8 $$\begin{aligned} J(d,\xi )=\sum (d-f(\xi ))^2, \end{aligned}$$

where $\xi$ represents the parameters we estimate, *d* is the set of data measurements, and $f(\xi )$ are the model output predictions. We perform validation by simulating the model with the estimated parameter values and calculating goodness of fit to the data measurements in the validation set.

For the control and *rd1* grouping case presented in the “Results” section, we also compute 95 % confidence interval (CI) for each estimated parameter by

9 $$\begin{aligned} {\hat{\xi }}\pm \left( t^{\alpha /2}_{m-p}\right) SE_{{\hat{\xi }}}, \end{aligned}$$

where ${\hat{\xi }}$ is the estimated parameter value, *m* is the number of data measurements, *p* is the number of estimated parameters, $t^{\alpha /2}_{m-p}$ is the statistic of the t-distribution with $m-p$ degrees of freedom at level of significance $\alpha$ (of 0.05), and $SE_{{\hat{\xi }}}$ is the standard error for the estimated parameter value. The standard errors are approximated by the square root of the diagonal elements of the covariance matrix for the estimated parameters

10 $$\begin{aligned} cov({\hat{\xi }})=s^2(L^{T}L)^{-1}. \end{aligned}$$

$s^2$ is the approximation of the variance of the data measurement errors, obtained by $s^2=J_{\text{ min }}/(m-p)$, where $J_{\text{ min }}$ is the minimum of the error function from the parameter estimation^61^. A confidence interval needs to be computed for each parameter estimate because this allows to obtain a range for the parameter estimate, which gives an indication about the uncertainty of the parameter value taking into account the quantity and variability of the data.

Additionally, we compute prediction intervals for the [GSH] and [GSSG] model outputs based on the estimated parameter values and compare these prediction intervals against all data measurements. The prediction intervals are computed with

11 $$\begin{aligned} \hat{y_{i}} \pm \left( t^{\alpha /2}_{m-p}\right) \sqrt{s^2[1+w_{i}^{T}(L^{T}L)^{-1}w_{i}]}, \end{aligned}$$

where $\hat{y_{i}}$ is the model response value and $w_{i}^{T}$ contains the values of the *i*th row of the sensitivity matrix *L* at the time for which the prediction interval is formed^61^. The quantities $t^{\alpha /2}_{m-p}$ and $s^2$ are as defined earlier.

#### Global sensitivity analysis (GSA)

GSA is a very useful tool to investigate the qualitative behavior of a mathematical model, and we have applied it previously to a model for cone photoreceptor metabolism^62,63^. In this study, GSA is used to examine which of the processes captured in the model for the GSH system are the most important in a control mouse retina compared to degenerative retina with RP. The information obtained from LSA aided in setting up and performing a more informed GSA. We conduct GSA with Partial Rank Correlation Coefficient (PRCC), which is a global method where all input factors (model parameters and/or initial conditions) are varied simultaneously using latin hypercube sampling. In latin hypercube sampling, the ranges over which parameters vary are specified and subdivided into regions of equal probability with no overlap between the regions. Next, an array of sample values is generated for each parameter by drawing a value from each region at random and without replacement. With these parameter values, model simulations are performed to collect information on an outcome of interest, which can be the model output or another outcome, and finally a PRCC is calculated for each input factor. PRCC quantifies the influence of a model input on the output of interest thus revealing the input factors that drive the largest changes in the level of the outcome of interest. An input factor is considered impactful if its corresponding PRCC value has a magnitude greater than some threshold usually set at 0.5, i.e., $|PRCC| > 0.5$, and the PRCC value is statistically significant with a p-value less than a specified significance level. In this study, we used a significance level of 0.001. A PRCC value could be negative, indicating that an input factor increase will cause the outcome of interest to decrease^64^.

## Results

### Parameters selected for estimation

Due to the amount of data available for [GSH] and [GSSG], two parameters could be estimated. Thus, only the most influential parameters were considered for estimation and included in the structured correlation analysis. In this study, we classified as most influential those parameters which have normalized local sensitivity coefficient $\frac{\partial y}{\partial \xi } \frac{\xi }{y}$ greater than a threshold of $10^{-1}$ for both [GSH] and [GSSG]. Also, the parameter set we analyze contains only influential parameters whose value could not be informed from the literature.

Based on the results from LSA and structured correlation analysis, $V_{\text{ max}_{{oxid}}}$ and $n_{\left[ \text{ GSSG }\right] }$ were the parameters selected for estimation; $V_{\text{ max}_{{oxid}}}$ is the maximum velocity of the reaction in which GSH becomes oxidized as it detoxifies ROS, and $n_{\left[ \text{ GSSG }\right] }$ reflects excretion of GSSG into extracellular space where it becomes hydrolyzed. As shown in Fig. 2, according to LSA in the control and the RP case, $V_{\text{ max}_{{oxid}}}$ and $n_{\left[ \text{ GSSG }\right] }$ are in the set of most influential parameters for both [GSH] and [GSSG]. The structured correlation analysis results presented in Table 1 show $V_{\text{ max}_{{oxid}}}$ and $n_{\left[ \text{ GSSG }\right] }$ to be identifiable since the magnitude of their pairwise correlation coefficient is less then 0.90 with value of 0.48 and 0.35 in the control mice and RP *rd1* mice, respectively. We can also see in Table 1 that the pair of parameters to estimate could not be $s_{\left[ \text{ GSH }\right] }$ and $n_{\left[ \text{ GSSG }\right] }$ because these parameters are correlated with the magnitude of their pairwise correlation coefficient higher than the threshold of 0.9 - equal to 0.96 and 0.97 for the control and RP case, respectively.

Among the possible choices of uncorrelated pairs, $V_{\text{ max}_{{oxid}}}$ and $n_{\left[ \text{ GSSG }\right] }$ are most suitable to estimate, as these parameters capture processes most directly related with the [GSH] and [GSSG] components for which we have the available experimental data.

**Table 1:** Structured correlation analysis results for the control and RP case.

Control								
	$s_{\left[ \text{ GSH }\right] }$	$\theta _{\left[ \text{ ROS }\right] }$	$V_{\text{ max}_{{oxid}}}$	*r*	$V_{p}$	$n_{\left[ \text{ GSSG }\right] }$		
$s_{\left[ \text{ GSH }\right] }$	1	− 0.07	0.54	0.53	− 0.06	0.96		
$\theta _{\left[ \text{ ROS }\right] }$		1	− 0.69	0.55	− 0.73	0.01		
$V_{\text{ max}_{{oxid}}}$			1	0.19	0.77	0.48		
*r*				1	-0.08	0.55		
$V_{p}$					1	−0.05		
$n_{\left[ \text{ GSSG }\right] }$						1		

RP								
	$s_{\left[ \text{ GSH }\right] }$	$n_{\left[ \text{ GSH }\right] }$	$\theta _{\left[ \text{ ROS }\right] }$	$V_{\text{ max}_{{oxid}}}$	*r*	$V_{p}$	$K_{n}$	$n_{\left[ \text{ GSSG }\right] }$
$s_{\left[ \text{ GSH }\right] }$	1	0.76	− 0.04	0.33	0.41	− 0.29	0.11	0.97
$n_{\left[ \text{ GSH }\right] }$		1	0.15	0.05	0.25	− 0.36	0.07	0.65
$\theta _{\left[ \text{ ROS }\right] }$			1	− 0.80	0.45	− 0.78	0.10	− 0.06
$V_{\text{ max}_{{oxid}}}$				1	0.15	0.77	-0.09	0.35
*r*					1	− 0.10	0.02	0.42
$V_{p}$						1	− 0.10	− 0.23
$K_{n}$							1	0.11
$n_{\left[ \text{ GSSG }\right] }$								1

### Calibration and validation results

As explained in the “Methods” section, we explored different grouping cases in forming the calibration and validation sets for the control and *rd1* data. We first fit the model to the calibration data, estimating the parameters $V_{\text{ max}_{{oxid}}}$ and $n_{\left[ \text{ GSSG }\right] }$. After that, in order to validate the model, we simulate the system of equations with the estimated parameter values and calculate goodness of fit of the [GSH] and [GSSG] simulation results to the validation set. The calibration and validation results for all cases are included in the Supplementary Information.

The initial values for [GSH] and [GSSG], i.e., $[\text{ GSH}](0)$ and $[\text{ GSSG}](0)$, respectively, are set to the initial point in their corresponding data set (at PN11), and the initial $[\text{ NADPH}]$ value, $[\text{ NADPH}](0)$, is informed by understanding that the GSH concentration is 2 to 3 orders of magnitude higher than the concentration of NADPH^28^. $[\text{ G6P}](0)$ is informed by experimental data from euglycemic Sprague-Dawley rat retinas^65^. For the control C3H mice, $[\text{ ROS}](0)$ is set to a very low level to reflect minor oxidative stress in control mouse retinas, and in the *rd1* case, $[\text{ ROS}](0)$ has a notably higher value to reflect increased oxidative stress.

In this section, for both control and *rd1* we present the grouping case with best outcome, that is high goodness of fit value for [GSH] and for [GSSG]. For control, the calibration set consists of the [GSH] and [GSSG] measurements at PN11, PN13 and PN15, and the validation set contains the data at PN15, PN17 and PN28. For *rd1*, the calibration set consists of the [GSH] and [GSSG] measurements at PN11, PN12 and PN13, and the validation set contains the data at PN15, PN17 and PN28.

**Figure 3:**
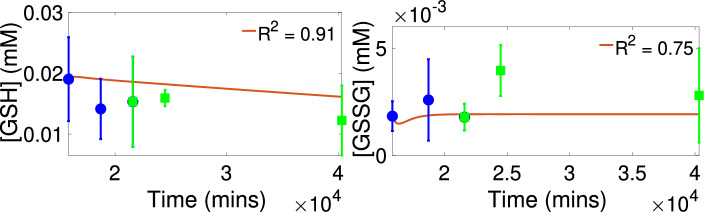
Model calibration and validation results with control data. Parameter values and initial conditions as given in Table 3. Calibration data shown with circles. Validation data shown with squares.

**Figure 4:**
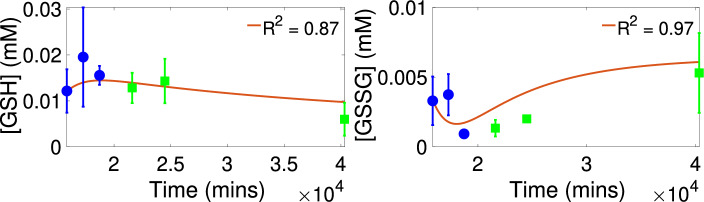
Model calibration and validation results with *rd1* data. Parameter values and initial conditions as given in Table 3. Calibration data shown with circles. Validation data shown with squares.

Figure 3 presents the calibration and validation results for control. We see that the model simulations with the estimated parameter values approximate the trends exhibited in the control validation data at PN15, PN17 and PN28 with goodness of fit $R^2=0.91$ for [GSH] and with $R^2=0.75$ for [GSSG]. In addition, the levels for [G6P], [ROS] and [NADPH] predicted by the model (see Fig. S1 in the Supplementary Information) are within physiological ranges. Figure 4 shows the calibration and validation results for the *rd1* case. We see that the model simulations with the estimated parameter values approximate the trends exhibited in the *rd1* validation data at PN15, PN17 and PN28 with goodness of fit $R^2=0.87$ for [GSH] and with $R^2=0.97$ for [GSSG]. Again, the levels for [G6P], [ROS] and [NADPH] predicted by the model (see Fig. S1 in the Supplementary Information) are within physiological ranges^28,65,66^. Table 2 presents the estimated values for the parameters $V_{\text{ max}_{{oxid}}}$ and $n_{\left[ \text{ GSSG }\right] }$ and their corresponding 95% confidence intervals. For both the control and the *rd1* case, the confidence intervals reveal notable level of uncertainty in these parameters. It should also be noted that the intervals contain the estimated values for these parameters obtained in the calibration and validation results for the other grouping set cases shown in the Supplementary Information.

**Figure 5:**
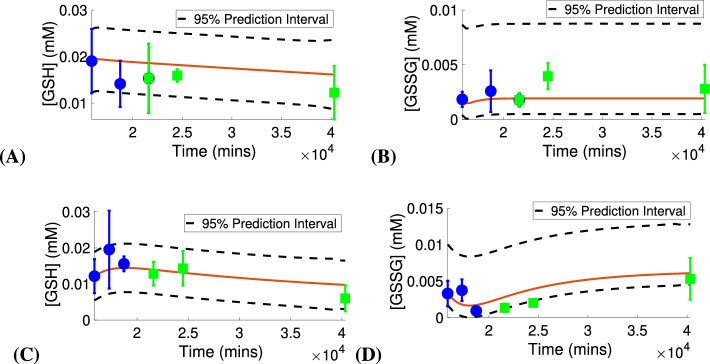
Prediction intervals for control (**A**, **B**) and RP conditions (**C**, **D**). Parameter values and initial conditions as given in Table 3. Calibration data shown with circles. Validation data shown with squares.

The results for the prediction intervals, computed based on the estimated parameter values from Table 2, are displayed in Fig. 5, and we see that both for the control and RP conditions, there is agreement between the prediction intervals for the model outputs [GSH] and [GSSG] and the data. The larger width of the prediction intervals is due to the noted parameter uncertainty.

**Table 2:** Confidence intervals for estimated parameter values.

Parameter	Control		*rd1*	
	Estimated value	95 % CI	Estimated value	95 % CI
$V_{\text{ max}_{{oxid}}}$	$1.6225 \times 10^{-1}$ mM$\cdot \text{ min}^{-1}$	($3.8344 \times 10^{-4}$, $5.3013 \times 10^{-1}$)	$7.5956 \times 10^{-3}$ mM$\cdot \text{ min}^{-1}$	($1.8315 \times 10^{-4}$, $3.0760 \times 10^{-2}$)
$n_{\left[ \text{ GSSG }\right] }$	$6.0728 \times 10^{-4}$ $\text{ min}^{-1}$	($2.4276 \times 10^{-7}$, $1.2143 \times 10^{-3}$)	$1.2526\times 10^{-4}$ $\text{ min}^{-1}$	($1.5106 \times 10^{-5}$, $1.6989\times 10^{-3}$)

**Table 3:** Parameter values and initial conditions (IC).

Parameter	Control		*rd1*	
	Value	References	Value	References
$s_{\left[ \text{ GSH }\right] }$	$1.1\times 10^{-6}$ mM$\cdot \text{ min}^{-1}$	Tuned	$1 \times 10^{-6}$ mM$\cdot \text{ min}^{-1}$	Tuned
$n_{\left[ \text{ NADPH }\right] }$	$1.3 \times 10^{-2}$ $\text{ min}^{-1}$	^49^	$1.3 \times 10^{-2}$ $\text{ min}^{-1}$	^49^
$n_{\left[ \text{ ROS }\right] }$	$1.67 \times 10^{-5}$ $\text{ min}^{-1}$	^49^	$1.67 \times 10^{-5}$ $\text{ min}^{-1}$	^49^
$n_{\left[ \text{ GSH }\right] }$	$2.7 \times 10^{-6}$ $\text{ min}^{-1}$	^49^	$3.5 \times 10^{-5}$ $\text{ min}^{-1}$	Tuned
$n_{\left[ \text{ GSSG }\right] }$	$6.0728 \times 10^{-4}$ $\text{ min}^{-1}$	Estimated	$1.2526\times 10^{-4}$ $\text{ min}^{-1}$	Estimated
$\theta _{\left[ \text{ ROS }\right] }$	0.95	Tuned	0.95	Tuned
$\theta _{\left[ \text{ NADPH }\right] }$	0.1	Tuned	0.1	Tuned
$V_{\text{ max}_{{reduc}}}$	$5\times 10^{-2}$ mM$\cdot \text{ min}^{-1}$	^67^	$5\times 10^{-2}$ mM$\cdot \text{ min}^{-1}$	^67^
$V_{\text{ max}_{{oxid}}}$	$1.6225 \times 10^{-1}$ mM$\cdot \text{ min}^{-1}$	Estimated	$7.5956 \times 10^{-3}$ mM$\cdot \text{ min}^{-1}$	Estimated
$K_{{s}_{\left[ \text{ GSSG }\right] }}$	5.05 mM	^49^	5.05 mM	^49^
$K_{{m}_{\left[ \text{ NADPH }\right] }}$	$7.8\times 10^{-3}$ mM	^67^	$7.8\times 10^{-3}$ mM	^67^
$K_{{m}_{\left[ \text{ GSSG }\right] }}$	$3.4\times 10^{-2}$ mM	^67^	$3.4\times 10^{-2}$ mM	^67^
$K_{{m}_{\left[ \text{ GSH }\right] }}$	0.2 mM	^49^	0.23 mM	Tuned
$K_{{m}_{\left[ \text{ ROS }\right] }}$	0.45 mM	^49^	0.45 mM	^49^
$K_{{s}_{\left[ \text{ ROS }\right] }}$	5.4 mM	^49^	5.4 mM	^49^
$\Phi$	0.76	^68^	0.76	^68^
*G*	3 mM	^68^	3 mM	^68^
*r*	$2\times 10^{-6}$ mM$\cdot \text{ min}^{-1}$	Tuned	$5.7 \times 10^{-6}$ mM$\cdot \text{ min}^{-1}$	Tuned
*q*	0.18	^68^	0.18	^68^
$\rho$	0.1	^69^	0.1	^69^
$\sigma _{\left[ \text{ GSH }\right] }$	1 mM$^{-1}$	Tuned	1 mM$^{-1}$	Tuned
$V_{p}$	$5\times 10^{-6}$ mM$\cdot \text{ min}^{-1}$	Tuned	$5\times 10^{-6}$ mM$\cdot \text{ min}^{-1}$	Tuned
$K_{p}$	$9\times 10^{-2}$ mM	^68^	$9\times 10^{-2}$ mM	^68^
$V_{f}$	$1\times 10^{-3}$ mM$\cdot \text{ min}^{-1}$	Tuned	$1\times 10^{-3}$ mM$\cdot \text{ min}^{-1}$	Tuned
$K_{f}$	0.1 mM	Tuned	0.1 mM	Tuned
$V_{n}$	1.5 mM$\cdot \text{ min}^{-1}$	Tuned	1.5 mM$\cdot \text{ min}^{-1}$	Tuned
$K_{n}$	$1\times 10^{-1}$ mM	Tuned	$1\times 10^{-1}$ mM	Tuned

IC	Control	*rd1*		
$[\text{ NADPH}](0)$	$1.9 \times 10^{-4}$ mM	$1.2 \times 10^{-4}$ mM		
$[\text{ ROS}](0)$	$10^{-8}$ mM	$10^{-4}$ mM		
$[\text{ GSH}](0)$	$1.9 \times 10^{-2}$ mM	$1.2 \times 10^{-2}$ mM		
$[\text{ GSSG}](0)$	$1.8 \times 10^{-3}$ mM	$2.5 \times 10^{-3}$ mM		
$[\text{ G6P}](0)$	$2 \times 10^{-4}$ mM	$2 \times 10^{-4}$ mM		

$^{*}$ Explanation on conversions of parameter values from references to the units used in this paper is provided in Table S1 in the Supplementary Information.

### GSA results

GSA is applied to more thoroughly investigate the model and to determine the most important processes in a control mouse retina compared to RP conditions. The input factors $V_{\text{ max}_{{reduc}}}$, $K_{{m}_{\left[ \text{ NADPH }\right] }}$, $K_{{m}_{\left[ \text{ GSSG }\right] }}$ were varied over ranges available from literature ([0.048, 0.056], [0.0076, 0.008], [0.032, 0.036], respectively^67^). The estimated parameters, $V_{\text{ max}_{{oxid}}}$ and $n_{\left[ \text{ GSSG }\right] }$ were varied over their corresponding 95 % confidence interval from Table 2. Due to unavailability of data, the remaining input factors were varied within 70% of their nominal values in Table 3.

**Figure 6:**
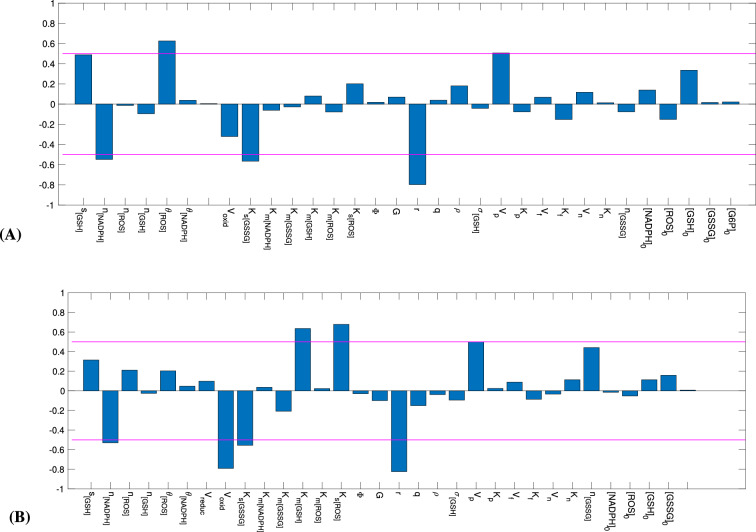
PRCC values with [GSH]/[GSSG] ratio at PN28 as outcome of interest for control (**A**) and RP case (**B**). (Input factors with PRCC values smaller than 0.01 in magnitude are omitted). From the parameters shown, those with |*PRCC*| > 0.5 are the influential ones included in Table 4. The lines in magenta are drawn at the values of − 0.5 and 0.5. For ease of readability, $V_{\text{ max}_{{oxid}}}$ is displayed as $V_{\text{ oxid }}$ and $V_{\text{ max}_{{reduc}}}$ is displayed as $V_{\text{ reduc }}$.

In this paper, we consider as outcomes of interest the [GSH]/[GSSG] ratio and the model outputs for the concentration of NADPH, ROS and G6P at time points for which calibration and validation data measurements are available (PN12, PN13, PN15, PN17, PN28). The [GSH]/[GSSG] ratio is used since it is considered an indicator of antioxidant capacity^37^. The GSA results, presented in Table 4, constitute the parameters deemed to be influential by PRCC, and the parameter lists are in decreasing order according to the magnitudes of the sensitivity measures. Input factors whose corresponding PRCC value is negative are designated with a negative sign in parentheses. The PRCC values for the influential input factors are shown in Table S3 in the Supplementary Information. Figure 6 displays the PRCC output with [GSH]/[GSSG] ratio at PN28 as outcome of interest for the control C3H mice and the RP *rd1* mice. From the input factors shown, those with |*PRCC*| > 0.5 are the ones included in Table 4.

#### [GSH]/[GSSG] ratio

The PRCC results show that for all postnatal days under control conditions and for PN28 under RP conditions, the [GSH]/[GSSG] ratio is most sensitive to changes in ROS creation due to photo-oxidation, represented by the parameter *r*. This parameter also has strong influence for the other postnatal days in the RP case. An increase in *r* makes the ratio decrease. This shows that in the postnatal period up to PN28 as more ROS is generated due to photo-oxidation, the retina experiences greater oxidative stress, as indicated by the [GSH]/[GSSG] ratio which has been documented in the literature^28^.

In RP conditions, the most influential parameter for [GSH]/[GSSG] on PN12 is $n_{\left[ \tiny \text{ GSSG }\right] }$ and on PN13, PN15, and PN17 the most influential parameter is $V_{\text{ max}_{{oxid}}}$. The parameter $V_{\text{ max}_{{oxid}}}$ also plays a role through the remaining postnatal days, and $n_{\left[ \tiny \text{ GSSG }\right] }$ has an impact at PN13, PN15 and PN17 as well. On the other hand, in control conditions, $n_{\left[ \tiny \text{ GSSG }\right] }$ does not appear as influential, and $V_{\text{ max}_{{oxid}}}$ exhibits importance in relation to the [GSH]/[GSSG] ratio only on PN12. The parameter $n_{\left[ \tiny \text{ GSSG }\right] }$ reflects cellular excretion of GSSG, and the corresponding PRCC value is positive. This means that during postnatal development in diseased retinas, greater excretion of GSSG, into extracellular space where it get hydrolyzed, could lead to an increase in the [GSH]/[GSSG] ratio. The parameter $V_{\text{ max}_{{oxid}}}$ represents the maximum velocity of the reaction in which GSH becomes oxidized as it neutralizes ROS. An increase in $V_{\text{ max}_{{oxid}}}$, which accelerates the oxidation of GSH to GSSG, leads to a decrease in the [GSH]/[GSSG] ratio.

In control conditions, $K_{\tiny {s}_{\left[ \tiny \text{ GSSG }\right] }}$ impacts the [GSH]/[GSSG] level for all postnatal days, except PN17, and in RP conditions, it is influential at PN28. The parameter $K_{\tiny {s}_{\left[ \tiny \text{ GSSG }\right] }}$ is a dissociation constant whose increased value would lower the rate of the reaction in which GSSG is reduced to GSH, thereby greater GSSG concentration would result leading to a lower [GSH]/[GSSG] ratio.

While in control retinas, the initial GSH amount $[\text{ GSH}]_{0}$ (at PN11) impacts the level of the [GSH]/[GSSG] ratio for all examined postnatal days, except PN28, in the RP case $[\text{ GSH}]_{0}$ does not appear to play a role. With $[\text{ GSH}]_{0}$, the initial GSH amount is denoted as an input factor in the sensitivity analysis to determine the impact of the initial GSH concentration on the system. Each of the values for this input factor, generated by the latin hypercube sampling, is used as an initial condition for GSH, $[\text{ GSH}](0)$, in starting the numerical simulation. So, this GSA finding suggests that in diseased retinas, the initial GSH level does not seem to be among the significant process driving the system.

Additionally, $\theta _{\left[ \text{ ROS }\right] }$ exhibits importance in relation to [GSH]/[GSSG] ratio only in the control case. The parameter $\theta _{\left[ \text{ ROS }\right] }$ is a modulating factor for the rate at which [ROS] decreases in response to the enzymatic reducing reaction of GSH. As indicated by the positive PRCC value, higher $\theta _{\left[ \text{ ROS }\right] }$ results in an increase in the [GSH]/[GSSG] ratio. This is because higher $\theta _{\left[ \text{ ROS }\right] }$ leads to a lower level of ROS, so less GSH gets used for detoxification, which in turn leads to an increase in the [GSH]/[GSSG] ratio.

On the other hand in RP, $K_{\tiny {s}_{\left[ \tiny \text{ ROS }\right] }}$ and $K_{\tiny {m}_{\left[ \tiny \text{ GSH }\right] }}$ are impactful for all postnatal days, but they do not appear as important in control conditions. The parameter $K_{\tiny {s}_{\left[ \tiny \text{ ROS }\right] }}$ represents a dissociation constant whose increased value would lower the rate of the detoxification reaction, causing less use of GSH and thus increased [GSH]/[GSSG] ratio. $K_{\tiny {m}_{\left[ \tiny \text{ GSH }\right] }}$ gives the GSH concentration necessary to achieve half the maximal velocity of the reaction in which GSH detoxifies ROS.

In addition, in RP conditions, the [GSH]/[GSSG] ratio is sensitive to changes in the parameter $n_{\left[ \tiny \text{ NADPH }\right] }$ at PN15, PN17 and PN28, in other words after the peak of rod degeneration. The ratio is impacted by this parameter on the same postnatal days in control conditions as well. Also, $V_{p}$ is influential at PN13, PN15, PN17 and PN28 in control retinas and at PN28 in RP retinas. The parameter $V_{p}$ is the maximum velocity of glucose conversion to G6P. Higher $V_{p}$ leads to a higher ratio due to larger production of G6P, and thereby more substrate for the synthesis of NADPH, which is necessary to convert GSSG back to GSH. The parameter $n_{\left[ \tiny \text{ NADPH }\right] }$ reflects the use of NADPH in processes other than the reduction of GSSG to GSH. An increase in $n_{\left[ \tiny \text{ NADPH }\right] }$ results in a lower ratio, as there is less NADPH available to return glutathione to its reduced state. These findings underscore the importance of NADPH in dealing with oxidative stress especially at times when most or all of the rods have degenerated, thereby exacerbating photo-oxidation due to increase of inflow of oxygen.

#### [NADPH]

In both control and RP conditions, the level of [NADPH] is sensitive to changes in $V_{p}$ and $n_{\left[ \tiny \text{ NADPH }\right] }$. Higher $V_{p}$ leads to greater production of G6P from glucose and thus results in an increase in the [NADPH] level. On the other hand, higher $n_{\left[ \tiny \text{ NADPH }\right] }$, which signifies diversion of NADPH to biosynthetic reactions, causes a decrease in its level.

#### [ROS]

For all postnatal days in the RP case and for PN15, PN17 and PN28 in the control case, changes in *r*, the parameter quantifying ROS creation due to photo-oxidation, drive the largest changes in the level of [ROS]. This indicates that as the retina matures or degenerates photo-oxidation becomes a main contributor to the generation and accumulation of ROS. Additionally, in both cases the parameters $K_{\tiny {m}_{\left[ \tiny \text{ GSH }\right] }}$ and $K_{\tiny {s}_{\left[ \tiny \text{ ROS }\right] }}$ are influential for PN12, PN13 and PN15. The ROS concentration is also impacted by $\theta _{\left[ \text{ ROS }\right] }$ and $V_{\text{ max}_{{oxid}}}$ for all postnatal days in control retinas, and in RP retinas these parameters have an impact at all postnatal days with the exception of PN12 for $\theta _{\left[ \text{ ROS }\right] }$ and PN28 for $V_{\text{ max}_{{oxid}}}$. This suggests that the process of GSH-mediated ROS detoxification has important influence on the level of [ROS] during retinal development and degeneration.

In control retinas, [ROS] is sensitive to changes in $[\text{ GSH}]_{0}$ for all postnatal days except PN28, and under RP conditions, [ROS] is sensitive to $[\text{ GSH}]_{0}$ at PN13, PN15 and PN17. On the other hand, $n_{\left[ \tiny \text{ GSSG }\right] }$ becomes influential at PN28 only in the RP case. Finally, in both control and RP conditions, $s_{\left[ \tiny \text{ GSH }\right] }$ exhibits importance in relation to the level of ROS at PN28. This suggests that the GSH production process and cellular excretion of GSSG are factors that would impact the level of [ROS] in diseased retinas after all rods have degenerated.

#### [G6P]

In control and in diseased retinas, the level of [G6P] is sensitive to changes in $V_{p}$, $V_{n}$, $K_{n}$, $\Phi$ for all postnatal days. An increase in $V_{p}$ results in higher [G6P]. $V_{n}$, $K_{n}$ and $\Phi$ are the parameters involved in the term that represents the diversion of G6P to the PPP for synthesizing NADPH. Increases in $\Phi$, the proportion of G6P diverted to the PPP, and $V_{n}$, the maximum rate of NADPH production from G6P, cause [G6P] to decrease. On the other hand, larger $K_{n}$, which is the half-limiting value of the NADPH maximum production rate, makes the [G6P] level increase.

**Table 4:**
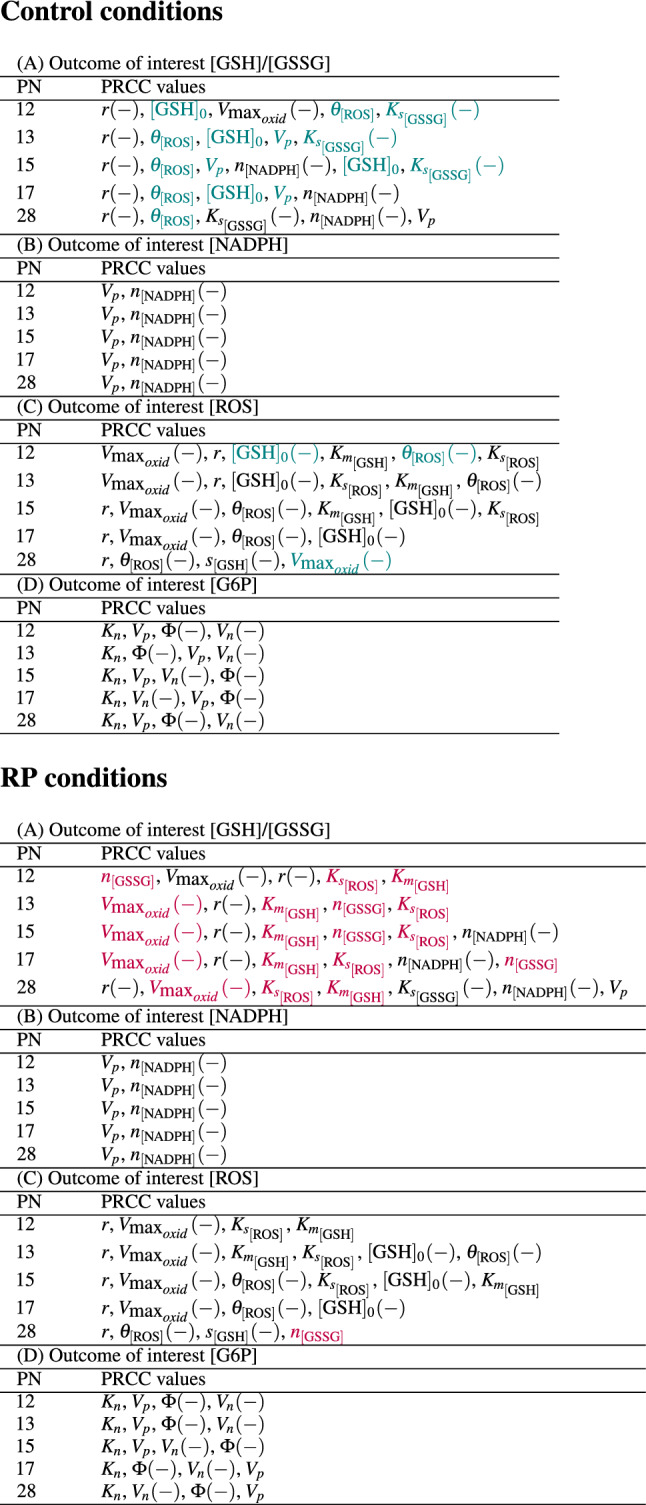
PRCCC results for the model calibrated and validated to data for control and RP conditions.

Colored in teal are input factors that are influential in the control but not in the RP retina. Colored in pink are input factors that are influential in the RP retina but not in the control. *PN* postnatal day.

## Discussion

In this paper, we develop the first mathematical model of the GSH antioxidant system in the outer retina, capturing the most essential components for ROS creation, GSH production, oxidation in neutralizing ROS, and reduction as well as the diversion of glucose into the PPP to synthesize NADPH. Applying LSA and structured correlation analysis, we identified influential and identifiable model parameters. From these, we selected for estimation parameters that were not available from literature and that are most directly associated with the dynamics of the GSH and GSSG concentrations ([GSH] and [GSSG], respectively), for which we had available experimental data. We performed model calibration through parameter estimation and subsequently validated the model. This was achieved using experimental [GSH] and [GSSG] data consisting of measurements at different postnatal days from PN11 up to PN28 from the retinas of control C3H mice and the *rd1* mouse model for the disease RP. The calibration and validation results show that both in the control and the *rd1* case, the model produces output for [GSH] and [GSSG] approximating trends in the experimental data, and there is notable uncertainty in the estimated parameters.

In order to better understand which pathways have the greatest impact on the system in control compared to RP retinas during the first 28 days of postnatal development, we use the GSA method PRCC to investigate the importance of model parameters and initial conditions in relation to the [GSH]/[GSSG] ratio, which reflects antioxidant capacity, as well as in relation to the concentrations of NADPH, ROS and G6P. The results show that overall in both control and diseased retinas, ROS generation due to photo-oxidation, quantified by *r*, drives large changes in the level of the [GSH]/[GSSG] ratio, exerting a negative effect, which indicates that this process is potentially a great contributor to increased oxidative stress.

In addition, in RP conditions the redox system is disturbed and as such for all postnatal days the [GSH]/[GSSG] ratio is sensitive to elements that govern the enzymatic reaction in which GSH reduces ROS; in particular to parameters representing the maximal velocity of the reaction ($V_{\text{ max}_{{oxid}}}$), the dissociation constant of ROS in this reaction ($K_{\tiny {s}_{\left[ \tiny \text{ ROS }\right] }}$), and concentration of GSH necessary to achieve half $V_{\text{ max}_{{oxid}}}$ when ROS is present at a saturating concentration ($K_{\tiny {m}_{\left[ \tiny \text{ GSH }\right] }}$). Furthermore, $V_{\text{ max}_{{oxid}}}$ is the most influential parameter at postnatal days 13, 15 and 17, which suggests that the maximum velocity of the GSH-mediated reaction to neutralize ROS has the greatest impact on oxidative stress in diseased retinas prior to degeneration of all rods, which occurs by PN20 in the *rd1* mouse model for RP^37^.

The PRCC analysis also shows that the process of ROS detoxification by GSH has strong effect on the level of [ROS] during the development of both healthy and diseased retinas. However, while in control retinas, the initial GSH concentration $[\text{ GSH}]_{0}$ (at PN11) impacts the level of [ROS] and the [GSH]/[GSSG] ratio for most postnatal days, in the RP case $[\text{ GSH}]_{0}$ does not appear to play a role in relation to the ratio, and in relation to the [ROS] level it has influence for three out of the five examined postnatal days. These findings suggest that the initial GSH concentration has a limited effect on antioxidant capacity and the level of [ROS] in RP retinas. Although, in RP retinas, and also in control ones, the [ROS] level is sensitive to the process of GSH production at PN28. This indicates that stimulating GSH production, which has been shown to prevent degeneration of RPE cells^70^, could potentially serve as an intervention strategy to combat ROS in maturing mouse retinas with RP where all rods have degenerated.

In addition, according to the PRCC results, in both control and RP retinas, higher utilization of NADPH in processes other than reducing oxidized glutathione, captured by the parameter $n_{\left[ \tiny \text{ NADPH }\right] }$, negatively affects the level of the [GSH]/[GSSG] ratio. This means that diverting a lot of NADPH to service other reactions would increase oxidative stress. It is key to note that for RP retinas, the sensitivity of the [GSH]/[GSSG] ratio to $n_{\left[ \tiny \text{ NADPH }\right] }$ emerges after the peak in rod degeneration, which occurs between PN11 and PN13 in the *rd1* mouse model for RP^37^. In addition to participating in detoxification reactions, NADPH is involved in the synthesis of lipids and proteins^71^, and in mice the retina fully matures until about PN30^72^ during which time it needs to sustain a high level of lipid and protein production^73^. In light of this, our finding points out that during the period of retinal maturation when there are significant structural maintenance demands requiring elevated lipid and protein synthesis, a careful balance needs to be maintained in the use of NADPH between antioxidant metabolism and biosynthetic processes. This is important for control retinas, but it is even more crucial for retinas with RP where the progressive loss of rods leads to increased oxygen tension and thereby exacerbated photo-oxidation.

Finally, in both control and RP conditions, the level of [NADPH] and the [GSH]/[GSSG] ratio are impacted by $V_{p}$, the maximum velocity of glucose conversion to G6P. Larger $V_{p}$ would result in greater synthesis of G6P from glucose, making more substrate available for diversion to the PPP to produce NADPH, which is required for the conversion of GSSG back to the reduced form GSH. In particular, for the RP case the [GSH]/[GSSG] ratio becomes impacted by the parameter $V_{p}$ at PN28, in other words after the death of all rods in the *rd1* mouse model for RP. Activating pathways for creation of NADPH has been shown to protect RPE cells from oxidative damage^74^, and our findings indicate that for developing mouse retinas with RP in which all rods have been lost a possible intervention strategy would be to stimulate synthesis of NADPH.

## Supplementary Information

Supplementary Information.

## Data Availability

Computer code for LSA, parameter estimation, numerical simulations, and GSA with PRCC was implemented in and run with the programming and computation software MATLAB (MathWorks Inc., Natick, MA, USA). The codes for LSA and numerical simulations use the MATLAB ODE solver routine *ode15s*. The code for the GSA method PRCC uses random number generation and statistics routines, and was modified and adapted from codes available at http://malthus.micro.med.umich.edu/lab/usadata/^64^. PRCC data for our model generated during the study is included in the Supplementary Information. No experimental datasets were generated during the current study; for model calibration and validation this manuscript used GSH and GSSG data collected as part of a previously published study (Gimeno-Hernández et al. (2020)) and unpublished data generated after that experimental study, but before the conception of this computational study. The data are included in the Supplementary Information.

## References

[1] Winkler BS, Boulton ME, Gottsch JD, Sternberg P (1999). Oxidative damage and age-related macular degeneration. Mol. Vis..

[2] Léveillard T, Philp NJ, Sennlaub F (2019). Is retinal metabolic dysfunction at the center of the pathogenesis of age-related macular degeneration?. Int. J. Mol. Sci..

[3] Bellezza I (2018). Oxidative stress in age-related macular degeneration: Nrf2 as therapeutic target. Front. Pharmacol..

[4] Ivanov IV, Mappes T, Schaupp P, Lappe C, Wahl S (2018). Ultraviolet radiation oxidative stress affects eye health. J. Biophoton..

[5] Léveillard T, Sahel J-A (2017). Metabolic and redox signaling in the retina. Cell. Mol. Life Sci..

[6] Yang M (2022). Expression of glucose transporter-2 in murine retina: Evidence for glucose transport from horizontal cells to photoreceptor synapses. J. Neurochem..

[7] Kanow MA (2017). Biochemical adaptations of the retina and retinal pigment epithelium support a metabolic ecosystem in the vertebrate eye. Elife.

[8] Jaroszynska N, Harding P, Moosajee M (2021). Metabolism in the zebrafish retina. J. Dev. Biol..

[9] Fu Z, Kern TS, Hellström A, Smith LEH (2021). Fatty acid oxidation and photoreceptor metabolic needs. J. Lipid Res..

[10] Joyal J-S (2016). Retinal lipid and glucose metabolism dictates angiogenesis through the lipid sensor ffar1. Nat. Med..

[11] Liu H, Prokosch V (2021). Energy metabolism in the inner retina in health and glaucoma. Int. J. Mol. Sci..

[12] Narayan DS, Chidlow G, Wood JPM, Casson RJ (2017). Glucose metabolism in mammalian photoreceptor inner and outer segments. Clin. Exp. Ophthalmol..

[13] Turrens JF (2003). Mitochondrial formation of reactive oxygen species. J. Physiol..

[14] Masuda T, Shimazawa M, Hara H (2017). Retinal diseases associated with oxidative stress and the effects of a free radical scavenger (edaravone). Oxid. Med. Cell. Longev..

[15] Nita M, Grzybowski A (2016). The role of the reactive oxygen species and oxidative stress in the pathomechanism of the age-related ocular diseases and other pathologies of the anterior and posterior eye segments in adults. Oxid. Med. Cell. Longev..

[16] Ren X, Léveillard T (2022). Modulating antioxidant systems as a therapeutic approach to retinal degeneration. Redox Biol..

[17] Keys SA, Zimmerman WF (1999). Antioxidant activity of retinol, glutathione, and taurine in bovine photoreceptor cell membranes. Exp. Eye Res..

[18] Chen Y (2012). Mechanism of all-trans-retinal toxicity with implications for stargardt disease and age-related macular degeneration. J. Biol. Chem..

[19] Hunter JJ (2012). The susceptibility of the retina to photochemical damage from visible light. Prog. Retin. Eye Res..

[20] van Norren D, Vos JJ (2016). Light damage to the retina: An historical approach. Eye (London).

[21] Chalam KV, Khetpal V, Rusovici R, Balaiya S (2011). A review: Role of ultraviolet radiation in age-related macular degeneration. Eye Contact Lens.

[22] Stone J (1999). Mechanisms of photoreceptor death and survival in mammalian retina. Prog. Retin. Eye Res..

[23] Elachouri G (2015). Thioredoxin rod-derived cone viability factor protects against photooxidative retinal damage. Free Radic. Biol. Med..

[24] Hunt RC, Handy I, Smith A (1996). Heme-mediated reactive oxygen species toxicity to retinal pigment epithelial cells is reduced by hemopexin. J. Cell. Physiol..

[25] Petit L (2018). Aerobic glycolysis is essential for normal rod function and controls secondary cone death in retinitis pigmentosa. Cell Rep..

[26] Deshmukh M, Kutscher H, Stein S, Sinko P (2009). Nonenzymatic, self-elimination degradation mechanism of glutathione. Chem. Biodiv..

[27] Balendiran GK, Dabur R, Fraser D (2004). The role of glutathione in cancer. Cell Biochem. Funct..

[28] Bradshaw PC (2019). Cytoplasmic and mitochondrial nadph-coupled redox systems in the regulation of aging. Nutrients.

[29] Punzo C, Kornacker K, Cepko C (2009). Stimulation of the insulin/mtor pathway delays cone death in a mouse model of retinitis pigmentosa. Nat. Neurosci..

[30] Adler L, Chen C, Koutalos Y (2014). Mitochondria contribute to nadph generation in mouse rod photoreceptors. J. Biol. Chem..

[31] Maier T, Leibundgut M, Ban N (2008). The crystal structure of a mammalian fatty acid synthase. Science.

[32] Miyazono S, Shimauchi-Matsukawa Y, Tachibanaki S, Kawamura S (2008). Highly efficient retinal metabolism in cones. Proc. Natl. Acad. Sci..

[33] Lu SC (2013). Glutathione synthesis. Biochem. Biophys. Acta..

[34] Townsend DM, Tew KD, Tapiero H (2003). The importance of glutathione in human disease. Biomed. Pharmacother..

[35] Cacciatore I, Cornacchia C, Pinnen F, Mollica A, Di Stefano A (2010). Prodrug approach for increasing cellular glutathione levels. Molecules.

[36] Ahuja-Jensen P (2007). Low glutathione peroxidase in rd1 mouse retina increases oxidative stress and proteases. NeuroRep..

[37] Gimeno-Hernández R (2020). Thioredoxin delays photoreceptor degeneration, oxidative and inflammation alterations in retinitis pigmentosa. Front. Pharmacol..

[38] Komalapriya C (2015). Integrative model of oxidative stress adaptation in the fungal pathogen candida albicans. PLoS ONE.

[39] Baudouin-Cornu P, Lagniel G, Kumar C, Huang M-E, Labarre J (2012). Glutathione degradation is a key determinant of glutathione homeostasis. J. Biol. Chem..

[40] Raftos JE, Whillier S, Kuchel PW (2010). Glutathione synthesis and turnover in the human erythrocyte. J. Biol. Chem..

[41] Reed MC (2008). A mathematical model of glutathione metabolism. Theor. Biol. Med. Model..

[42] Nijhout HF (2009). A mathematical model gives insights into the effects of vitamin b-6 deficiency on 1-carbon and glutathione metabolism. J. Nutr..

[43] Lawley SD (2014). Mathematical modeling of the effects of glutathione on arsenic methylation. Theor. Biol. Med. Model..

[44] Geenen S (2013). Glutathione metabolism modeling: a mechanism for liver drug-robustness and a new biomarker strategy. Biochem. Biophys. Acta..

[45] Pannala VR, Bazil JN, Camara AKS, Dash RK (2013). A biophysically based mathematical model for the catalytic mechanism of glutathione reductase. Free Radic. Biol. Med..

[46] Pannala VR, Bazil JN, Camara AKS, Dash RK (2014). A mechanistic mathematical model for the catalytic action of glutathione peroxidase. Free Radic. Res..

[47] Kagan VE (2017). Oxidized arachidonic and adrenic pes navigate cells to ferroptosis. Nat. Chem. Biol..

[48] Adimora NJ, Jones DP, Kemp ML (2010). A model of redox kinetics implicates the thiol proteome in cellular hydrogen peroxide responses. Antioxid. Redox Signal..

[49] Bhowmick R, Sarkar RR (2020). Differential suitability of reactive oxygen species and the role of glutathione in regulating paradoxical behavior in gliomas: A mathematical perspective. PLoS ONE.

[50] Roy M, Finley SD (2017). Computational model predicts the effects of targeting cellular metabolism in pancreatic cancer. Front. Physiol..

[51] Lushchak VI (2012). Glutathione homeostasis and functions: Potential targets for medical interventions. J. Amino Acids.

[52] Voet, D. & Voet, J. G. *Biochemistry*, chap. 14: Rates of enyzmatic reactions, 4th edn. 482–505 (Wiley, 2010),

[53] Purich, D. L. *Enzyme Kinetics: Catalysis & Control*, chap. 6: Initial-rate kinetics of multi-substrate enzyme-catalyzed reactions, 335–373 (Elsevier, 2010).

[54] Mei X (2016). The thioredoxin encoded by the rod-derived cone viability factor gene protects cone photoreceptors against oxidative stress. Antioxid. Redox Signal..

[55] Dickinson RP, Gelinas RJ (1976). Sensitivity analysis of ordinary differential equation systems—a direct method. J. Comput. Phys..

[56] Eslami, M. *Theory of Sensitivity in Dynamic Systems*, chap. 2: The principal aspects of sensitivity theory, 17–73 (Springer Berlin, 1994).

[57] Saltelli, A., Chan, K. & Scott, E. M. (eds.). *Sensitivity Analysis*, chap. Local methods, 81–99 (John Wiley & Sons, 2000).

[58] Olufsen MS, Ottesen JT (2013). A practical approach to parameter estimation applied to model predicting heart rate regulation. J. Math. Biol..

[59] Reed DJ (1980). High-performance liquid chromatography analysis of nanomole levels of glutathione, glutathione disulfide, and related thiols and disulfides. Anal. Biochem..

[60] Lowry OH, Rosebrough NJ, Farr AL, Randall RJ (1951). Protein measurement with the folin phenol reagent. J. Biol. Chem..

[61] Smith RC (2014). Uncertainty Quantification: Theory, Implementation, and Applications.

[62] Dobreva A (2022). Insights into pathological mechanisms and interventions revealed by analyzing a mathematical model for cone metabolism. Bioscience Reports.

[63] Camacho, E. T. *et al.* Mathematical modeling of retinal degeneration: Aerobic glycolysis in a single cone. In *Using Mathematics to Understand Biological Complexity*, 135–178 (Springer, 2020).

[64] Marino S, Hogue IB, Ray CJ, Kirschner DE (2008). A methodology for performing global uncertainty and sensitivity analysis in systems biology. J. Theor. Biol..

[65] Ola MS (2006). Analysis of glucose metabolism in diabetic rat retinas. Am. J. Physiol. Endocrinol. Metab..

[66] Mackey AM (2008). Redox survival signalling in retina-derived 661w cells. Cell Death Differ..

[67] Hsu SC, Molday RS (1994). Glucose metabolism in photoreceptor outer segments. Its role in phototransduction and in nadph-requiring reactions. J. Biol. Chem..

[68] Camacho ET (2019). A mathematical analysis of aerobic glycolysis triggered by glucose uptake in cones. Sci. Rep..

[69] Grenell A (2019). Loss of mpc1 reprograms retinal metabolism to impair visual function. Proc. Natl. Acad. Sci..

[70] Schimel AM (2011). N-acetylcysteine amide (naca) prevents retinal degeneration by up-regulating reduced glutathione production and reversing lipid peroxidation. Am. J. Pathol..

[71] Rajala R (2020). Aerobic glycolysis in the retina: Functional roles of pyruvate kinase isoforms. Front. Cell Dev. Biol..

[72] Völkner M (2021). Mouse retinal organoid growth and maintenance in longer-term culture. Front. Cell Dev. Biol..

[73] Rao SR, Fliesler SJ (2021). Cholesterol homeostasis in the vertebrate retina: Biology and pathobiology. J. Lipid Res..

[74] Yam M (2019). Proline mediates metabolic communication between retinal pigment epithelial cells and the retina. J. Biol. Chem..

