# Supplementary material for: Mathematical model for glutathione dynamics in the retina
Source: Sci Rep. 2023 Jul 7;13:10996. doi: 10.1038/s41598-023-37938-9 (PMC10328985; doi:10.1038/s41598-023-37938-9)
Supplement: Supplementary file 1 — Supplementary Information. [file 41598_2023_37938_MOESM1_ESM.pdf]

- Supplementary Information -

Mathematical model for glutathione dynamics in the retina

[1,*] Atanaska Dobreva, [2]Erika Tatiana Camacho, and [3]María Miranda

[1]Augusta University, Department of Mathematics, Augusta, GA, 30912, USA

[2]Arizona State University, School of Mathematical and Statistical Sciences, Tempe, AZ, 85281, USA

[3]Department of Biomedical Sciences, Faculty of Health Sciences, Institute of Biomedical Sciences, Cardenal Herrera-CEU University, CEU Universities, 46115 Valencia, Spain

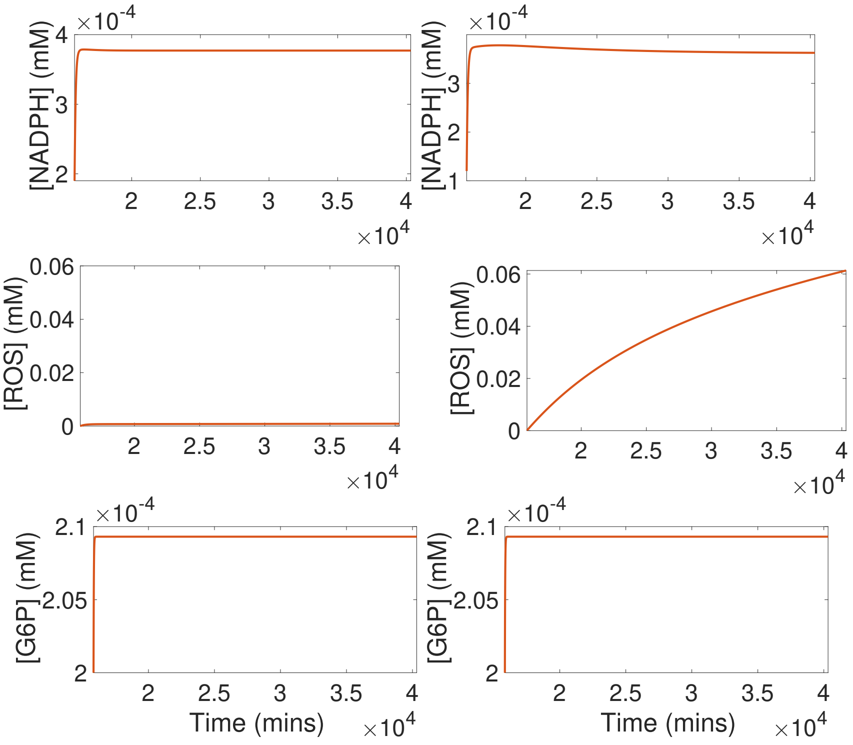

Figure S1: Model predictions for the concentrations of G6P, ROS and NADPH for control case (left panels) and *rd1* case (right panels). Parameter values and initial conditions as given in Table 3 in the manuscript.

Table S1: Conversions for parameter values. mg: mg of protein

Parameter	Source value	Conversion factor	Converted value	Source
$V\max_{reduc}$	$(6 - 7)\times10^{-3}$ μmol$\cdot$min$^{-1}\cdot$ mg^{-1}	8 mg$\cdot$ml^{-1} (protein concentration)	$(4.8 - 5.6)\times10^{-2}$ mM$\cdot$min^{-1}	[65]
$K_{m_{[\mathrm{NADPH}]}}$	7.8 μM	10^{-3} mM $\cdot$ 1 μM^{-1}	7.8×10^{-3} mM	[65]
$K_{m_{[\mathrm{GSSG}]}}$	34 μM	10^{-3} mM $\cdot$ 1 μM^{-1}	3.4×10^{-2} mM	[65]

Below is the calculation to obtain the converted value range of $(4.8 - 5.6)\times10^{-2}$ mM$\cdot$min^{-1} for $V\max_{reduc}$ from the source value range of $(6 - 7)\times10^{-3}$ μmol$\cdot$min$^{-1}\cdot$ mg^{-1}.

$$\left((6-7)\times10^{-3}\frac{\mu\mathrm{mol}}{\mathrm{min}\cdot\mathrm{mg}}\right)\left(\frac{10^{-3}\mathrm{mmol}}{1\mu\mathrm{mol}}\right)\left(8\frac{\mathrm{mg}}{\mathrm{ml}}\right)\left(\frac{1000\mathrm{ml}}{1\mathrm{L}}\right)=(4.8-5.6)\times10^{-2}\frac{\mathrm{mmol}}{\mathrm{L}\cdot\mathrm{min}}$$
$$=(4.8-5.6)\times10^{-2}\ \mathrm{mM}\cdot\mathrm{min}^{-1}$$

Note that the value of 5×10^{-2} mM$\cdot$min^{-1} for $V\max_{reduc}$ in Table 3 in the manuscript is within the range $(4.8 - 5.6)\times10^{-2}$ mM$\cdot$min^{-1}.

Covariance matrix for structured correlation analysis

As explained in the paper, only the most influential parameters were considered for estimation and included in the structured correlation analysis for which covariance matrix C needed to be computed. We provide as an example how the covariance matrix C was computed for the control case.

We have $C = (L^T L)^{-1}$, where

$$L = \begin{bmatrix}
\frac{\partial[NADPH]}{\partial s_{\text{[GSH]}}}(t_1) & \frac{\partial[NADPH]}{\partial \theta_{\text{[ROS]}}}(t_1) & \frac{\partial[NADPH]}{\partial \text{Vmax}_{oxid}}(t_1) & \frac{\partial[NADPH]}{\partial r}(t_1) & \frac{\partial[NADPH]}{\partial V_p}(t_1) & \frac{\partial[NADPH]}{\partial n_{\text{[GSSG]}}}(t_1) \\
\vdots & \vdots & \vdots & \vdots & \vdots & \vdots \\
\frac{\partial[NADPH]}{\partial s_{\text{[GSH]}}}(t_N) & \frac{\partial[NADPH]}{\partial \theta_{\text{[ROS]}}}(t_N) & \frac{\partial[NADPH]}{\partial \text{Vmax}_{oxid}}(t_N) & \frac{\partial[NADPH]}{\partial r}(t_N) & \frac{\partial[NADPH]}{\partial V_p}(t_N) & \frac{\partial[NADPH]}{\partial n_{\text{[GSSG]}}}(t_N) \\
\frac{\partial[ROS]}{\partial s_{\text{[GSH]}}}(t_1) & \frac{\partial[ROS]}{\partial \theta_{\text{[ROS]}}}(t_1) & \frac{\partial[ROS]}{\partial \text{Vmax}_{oxid}}(t_1) & \frac{\partial[ROS]}{\partial r}(t_1) & \frac{\partial[ROS]}{\partial V_p}(t_1) & \frac{\partial[ROS]}{\partial n_{\text{[GSSG]}}}(t_1) \\
\vdots & \vdots & \vdots & \vdots & \vdots & \vdots \\
\frac{\partial[ROS]}{\partial s_{\text{[GSH]}}}(t_N) & \frac{\partial[ROS]}{\partial \theta_{\text{[ROS]}}}(t_N) & \frac{\partial[ROS]}{\partial \text{Vmax}_{oxid}}(t_N) & \frac{\partial[ROS]}{\partial r}(t_N) & \frac{\partial[ROS]}{\partial V_p}(t_N) & \frac{\partial[ROS]}{\partial n_{\text{[GSSG]}}}(t_N) \\
\frac{\partial[GSH]}{\partial s_{\text{[GSH]}}}(t_1) & \frac{\partial[GSH]}{\partial \theta_{\text{[ROS]}}}(t_1) & \frac{\partial[GSH]}{\partial \text{Vmax}_{oxid}}(t_1) & \frac{\partial[GSH]}{\partial r}(t_1) & \frac{\partial[GSH]}{\partial V_p}(t_1) & \frac{\partial[GSH]}{\partial n_{\text{[GSSG]}}}(t_1) \\
\vdots & \vdots & \vdots & \vdots & \vdots & \vdots \\
\frac{\partial[GSH]}{\partial s_{\text{[GSH]}}}(t_N) & \frac{\partial[GSH]}{\partial \theta_{\text{[ROS]}}}(t_N) & \frac{\partial[GSH]}{\partial \text{Vmax}_{oxid}}(t_N) & \frac{\partial[GSH]}{\partial r}(t_N) & \frac{\partial[GSH]}{\partial V_p}(t_N) & \frac{\partial[GSH]}{\partial n_{\text{[GSSG]}}}(t_N) \\
\frac{\partial[GSSG]}{\partial s_{\text{[GSH]}}}(t_1) & \frac{\partial[GSSG]}{\partial \theta_{\text{[ROS]}}}(t_1) & \frac{\partial[GSSG]}{\partial \text{Vmax}_{oxid}}(t_1) & \frac{\partial[GSSG]}{\partial r}(t_1) & \frac{\partial[GSSG]}{\partial V_p}(t_1) & \frac{\partial[GSSG]}{\partial n_{\text{[GSSG]}}}(t_1) \\
\vdots & \vdots & \vdots & \vdots & \vdots & \vdots \\
\frac{\partial[GSSG]}{\partial s_{\text{[GSH]}}}(t_N) & \frac{\partial[GSSG]}{\partial \theta_{\text{[ROS]}}}(t_N) & \frac{\partial[GSSG]}{\partial \text{Vmax}_{oxid}}(t_N) & \frac{\partial[GSSG]}{\partial r}(t_N) & \frac{\partial[GSSG]}{\partial V_p}(t_N) & \frac{\partial[GSSG]}{\partial n_{\text{[GSSG]}}}(t_N) \\
\frac{\partial[G6P]}{\partial s_{\text{[GSH]}}}(t_1) & \frac{\partial[G6P]}{\partial \theta_{\text{[ROS]}}}(t_1) & \frac{\partial[G6P]}{\partial \text{Vmax}_{oxid}}(t_1) & \frac{\partial[G6P]}{\partial r}(t_1) & \frac{\partial[G6P]}{\partial V_p}(t_1) & \frac{\partial[G6P]}{\partial n_{\text{[GSSG]}}}(t_1) \\
\vdots & \vdots & \vdots & \vdots & \vdots & \vdots \\
\frac{\partial[G6P]}{\partial s_{\text{[GSH]}}}(t_N) & \frac{\partial[G6P]}{\partial \theta_{\text{[ROS]}}}(t_N) & \frac{\partial[G6P]}{\partial \text{Vmax}_{oxid}}(t_N) & \frac{\partial[G6P]}{\partial r}(t_N) & \frac{\partial[G6P]}{\partial V_p}(t_N) & \frac{\partial[G6P]}{\partial n_{\text{[GSSG]}}}(t_N)
\end{bmatrix}$$

The form of C is provided on the next page.

$$C = \begin{bmatrix} Var(s_{[\mathrm{GSH}]}) & Cov(s_{[\mathrm{GSH}]}, \theta_{[\mathrm{ROS}]}) & Cov(s_{[\mathrm{GSH}]}, V_{\max_{oxid}}) & Cov(s_{[\mathrm{GSH}]}, r) & Cov(s_{[\mathrm{GSH}]}, V_p) & Cov(s_{[\mathrm{GSH}]}, n_{[\mathrm{GSSG}]}) \\ Cov(\theta_{[\mathrm{ROS}]}, s_{[\mathrm{GSH}]}) & Var(\theta_{[\mathrm{ROS}]}) & Cov(\theta_{[\mathrm{ROS}]}, V_{\max_{oxid}}) & Cov(\theta_{[\mathrm{ROS}]}, r) & Cov(\theta_{[\mathrm{ROS}]}, V_p) & Cov(\theta_{[\mathrm{ROS}]}, n_{[\mathrm{GSSG}]}) \\ Cov(V_{\max_{oxid}}, s_{[\mathrm{GSH}]}) & Cov(V_{\max_{oxid}}, \theta_{[\mathrm{ROS}]}) & Var(V_{\max_{oxid}}) & Cov(V_{\max_{oxid}}, r) & Cov(V_{\max_{oxid}}, V_p) & Cov(V_{\max_{oxid}}, n_{[\mathrm{GSSG}]}) \\ Cov(r, s_{[\mathrm{GSH}]}) & Cov(r, \theta_{[\mathrm{ROS}]}) & Cov(r, V_{\max_{oxid}}) & Var(r) & Cov(r, V_p) & Cov(r, n_{[\mathrm{GSSG}]}) \\ Cov(V_p, s_{[\mathrm{GSH}]}) & Cov(V_p, \theta_{[\mathrm{ROS}]}) & Cov(V_p, V_{\max_{oxid}}) & Cov(V_p, r) & Var(V_p) & Cov(V_p, n_{[\mathrm{GSSG}]}) \\ Cov(n_{[\mathrm{GSSG}]}, s_{[\mathrm{GSH}]}) & Cov(n_{[\mathrm{GSSG}]}, \theta_{[\mathrm{ROS}]}) & Cov(n_{[\mathrm{GSSG}]}, V_{\max_{oxid}}) & Cov(n_{[\mathrm{GSSG}]}, r) & Cov(n_{[\mathrm{GSSG}]}, V_p) & Var(n_{[\mathrm{GSSG}]}) \end{bmatrix}$$

Calculations for data values

To obtain the data values plotted in Figures 3, 4 and 5 in the manuscript, for each postnatal day (PN), we first converted the measurements with units of nmol/mg in Tables S2 and S3 to units of mM, using the following calculation, where the conversion factor is the protein concentration. We use as an illustrative example the first entry for GSH for CONTROL PN11 (56.35205697 nmol/mg) for which the protein concentration is 0.493 mg/ml.

$$\left(56.35205697\frac{\text{nmol}}{\text{mg}}\right)\left(\frac{10^{-6}\text{mmol}}{1\text{nmol}}\right)\left(0.493\frac{\text{mg}}{\text{ml}}\right)\left(\frac{10^{3}\text{ml}}{1\text{L}}\right) = 0.02778156\frac{\text{mmol}}{\text{L}} = 0.02778156 \text{ mM}$$

For each PN, after we converted from nmol/mg to mM, we calculated the average and the standard deviation. The standard deviations correspond to the error bars plotted for the data points in Figures 3, 4 and 5 in the manuscript.

Table S2: Experimental Data

AVE: average; SD: standard deviation; prot: protein concentration

GSH

CONTROL PN11 (nmol/mg prot)	prot (mg/ml)	CONTROL PN11 (mM)	
56.35205697	0.493	0.02778156	
31.99012957	0.652	0.02085756	
34.72830497	0.598	0.02076753	
38.74836	0.451685393	0.01750207	
30.77253	0.362921348	0.01116801	
25.04869	0.168539326	0.00422169	
51.75038	0.556179775	0.02878251	
36.82613	0.605617978	0.02230257	
43.6042212	0.434	0.01892423	
40.97948815	0.422	0.01729334	
47.91346602	0.412	0.01974035	
		0.01903104	AVE
		0.00689734	SD

CONTROL PN13 (nmol/mg prot)	prot (mg/ml)	CONTROL PN13 (mM)	
20.35201881	0.505	0.01027777	
39.87730404	0.495	0.01973927	
26.12796263	0.475	0.01241078	
		0.01414261	AVE
		0.00496280	SD

CONTROL PN15 (nmol/mg prot)	prot (mg/ml)	CONTROL PN15 (mM)	
67.17826228	0.375280899	0.02521072	
75.67878696	0.266292135	0.02015267	
54.06028968	0.393258427	0.02125966	
72.14148438	0.293258427	0.02115610	
17.41657627	0.48876541	0.00851262	
16.99639858	0.43542312	0.00740062	
16.30869148	0.39878938	0.00650373	
24.88229979	0.50023211	0.01244693	
		0.01533038	AVE
		0.00742157	SD

CONTROL PN 17 (nmol/mg prot)	prot (mg/ml)	CONTROL PN17 (mM)	
23.27909328	0.717	0.01668557	
24.63858114	0.664	0.01636002	
25.20093053	0.55	0.01386051	
25.3649335	0.577	0.01463557	
23.36597003	0.730526316	0.01706946	
21.7413357	0.781403509	0.01698876	
		0.01593331	AVE
		0.00135155	SD

CONTROL PN28 (nmol/mg prot)	prot (mg/ml)	CONTROL PN28 (mM)	
16.33783	0.485747126	0.00793605	
11.03925	0.731724138	0.00807769	
28.89157	0.706436782	0.02041007	
19.22188	0.459310345	0.00882881	
14.90766	0.631724138	0.00941753	
24.25293	0.775402299	0.01880578	
		0.01224599	AVE
		0.00574997	SD

GSSG

CONTROL PN11 (nmol/mg prot)	prot (mg/ml)	CONTROL PN11 (mM)	
5.67	0.45	0.00256106	
7.06	0.36	0.00256222	
6.45	0.17	0.00108687	
2.38	0.56	0.00132160	
2.68	0.61	0.00162319	
		0.00183099	AVE
		0.00069355	SD

CONTROL PN13 (nmol/mg prot)	prot (mg/ml)	CONTROL PN13 (mM)	
1.345236158	0.505	0.00067934	
5.259937374	0.495	0.00260367	
9.4216	0.475	0.00447526	
		0.00258609	AVE
		0.00189802	SD

CONTROL PN15 (nmol/mg prot)	prot (mg/ml)	CONTROL PN15 (mM)	
2.254398098	0.293258427	0.00066112	
5.308170187	0.375280899	0.00199205	
5.837000704	0.266292135	0.00155435	
6.398072963	0.393258427	0.00251610	
3.644628824	0.293258427	0.00106882	
		0.00155849	AVE
		0.00073315	SD

CONTROL PN17 (nmol/mg prot)	prot (mg/ml)	CONTROL PN17 (mM)	
6.489051621	0.717	0.00465111	
7.918447603	0.664	0.00526087	
5.150830736	0.550	0.00283345	
5.335057213	0.577	0.00307706	
		0.00395562	AVE
		0.00118582	SD

CONTROL PN28 (nmol/mg prot)	prot (mg/ml)	CONTROL PN28 (mM)	
7.19	0.4857471	0.00349336	
8.50	0.73	0.00622031	
4.78	0.71	0.00337844	
6.28	0.46	0.00288459	
1.02	0.63	0.00064556	
0.20	0.78	0.00015132	
		0.00279560	AVE
		0.00219900	SD

GSH

RD1 PN11 (nmol/mg prot)	prot (mg/ml)	RD1 PN11 (mM)	
30.93524233	0.596	0.01843740	
34.21058105	0.516	0.01765266	
39.33411211	0.514	0.02021773	
26.12486041	0.594	0.01551817	
24.28682158	0.2377736	0.00577477	
21.98799054	0.3391392	0.00745699	
14.82716585	0.5837388	0.00865519	
14.86080045	0.2928636	0.00435219	
14.71716091	0.599164	0.00881799	
16.15811115	0.5176308	0.00836394	
49.45603859	0.293965517	0.01453837	
16.92971164	0.380172414	0.00643621	
38.10231388	0.353448276	0.01346720	
23.65985575	0.270689655	0.00640448	
35.40815859	0.454	0.01607530	
35.32531579	0.456	0.01610834	
35.10195652	0.46	0.01614690	
		0.01202493	AVE
		0.00516751	SD

RD1 PN12 (nmol/mg prot)	prot (mg/ml)	RD1 PN12 (mM)	
37.22233432	0.95154	0.03541854	
16.29462568	1.380864	0.02250066	
23.04965285	1.51375	0.03489141	
47.62076306	0.325	0.01547675	
36.0427448	0.405	0.01459731	
12.51773152	0.3825	0.00478803	
24.50959542	0.5075	0.01243862	
51.7887812	0.3125	0.01618399	
		0.01953691	AVE
		0.01080220	SD

RD1 PN13 (nmol/mg prot)	prot (mg/ml)	RD1 PN13 (mM)	
38.77452436	0.431	0.01671182	
31.99166262	0.412	0.01318056	
33.49701896	0.501	0.01678201	
		0.01555813	AVE
		0.00205933	SD

RD1 PN15 (nmol/mg prot)	prot (mg/ml)	RD1 PN15 (mM)	
33.36116181	0.36	0.01201002	
47.37113401	0.347	0.01643778	
30.83263769	0.325	0.01002061	
		0.01282280	AVE
		0.00328489	SD

RD1 PN17 (nmol/mg prot)	prot (mg/ml)	RD1 PN17 (mM)	
36.46272078	0.41	0.01494972	
29.60759546	0.529	0.01566242	
32.63169119	0.436	0.01422742	
13.24907134	0.380172414	0.00503693	
32.6188807	0.362068966	0.01181028	
41.32130123	0.393103448	0.01624355	
51.79074904	0.405172414	0.02098418	
14.57032652	0.390517241	0.00568996	
50.30986508	0.399137931	0.02008058	
33.15332599	0.477894737	0.01584380	
41.58681894	0.377894737	0.01571544	
36.58049026	0.432280702	0.01581304	
		0.01433811	AVE
		0.00483311	SD

RD1 PN28 (nmol/mg prot)	prot (mg/ml)	RD1 PN28 (mM)	
9.5226697	0.422970297	0.00402781	
2.88290985	0.448712871	0.00129360	
28.4450825	0.345742574	0.00983468	
22.9261073	0.403168317	0.00924308	
15.8664968	0.353663366	0.00561140	
		0.00600211	AVE
		0.00358514	SD

GSSG

RD1 PN11 (nmol/mg prot)	prot (mg/ml)	RD1 PN11 (mM)	
10.23	0.5837388	0.00597303	
4.51	0.27	0.00122031	
5.51	0.35	0.00194874	
2.49	0.38	0.00094555	
		0.00252191	AVE
		0.00233936	SD

RD1 PN12 (nmol/mg prot)	prot (mg/ml)	RD1 PN12 (mM)	
5.492405994	0.95154	0.00522624	
1.639851571	1.380864	0.00226441	
2.471433196	1.51375	0.00374113	
		0.00374393	AVE
		0.00148092	SD

RD1 PN 13 (nmol/mg prot)	prot (mg/ml)	RD1 PN13 (mM)	
2.372385151	0.431	0.00102250	
2.468706311	0.412	0.00101711	
1.492005988	0.501	0.00074749	
		0.00092903	AVE
		0.00015724	SD

RD1 PN 15 (nmol/mg prot)	prot (mg/ml)	RD1 PN15 (mM)	
3.337988889	0.36	0.00120168	
2.393291066	0.347	0.00083047	
6.112446154	0.325	0.00198655	
		0.00133956	AVE
		0.00059024	SD

RD1 PN17 (nmol/mg prot)	prot (mg/ml)	RD1 17 (mM)	
4.302444284	0.477894737	0.00205612	
5.240374652	0.377894737	0.00198031	
4.577761607	0.432280702	0.00197888	
		0.00200510	AVE
		0.00004419	SD

RD1 PN28 (nmol/mg prot)	prot (mg/ml)	RD1 PN28 (mM)	
9.321553181	0.4229703	0.00394274	
22.17338982	0.45	0.00994949	
16.52557479	0.35	0.00571359	
5.879444039	0.40	0.00237041	
12.81895579	0.35	0.00453360	
		0.00530196	AVE
		0.00286360	SD

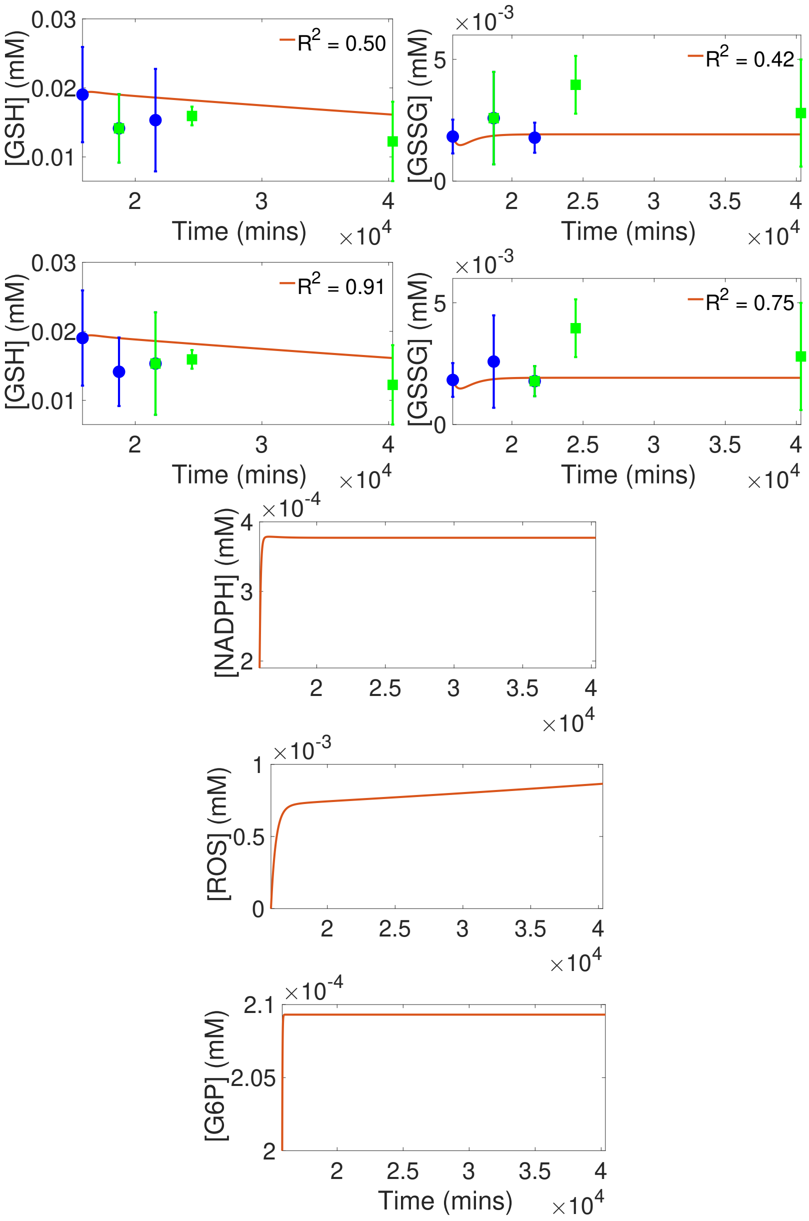

Figure S2: Model calibration and validation for control - case A. $V_{\max_{oxid}} = 1.6225 \times 10^{-1}$, $n_{[\text{GSSG}]} = 6.0728 \times 10^{-4}$. Calibration data shown with circles. Validation data shown with squares.

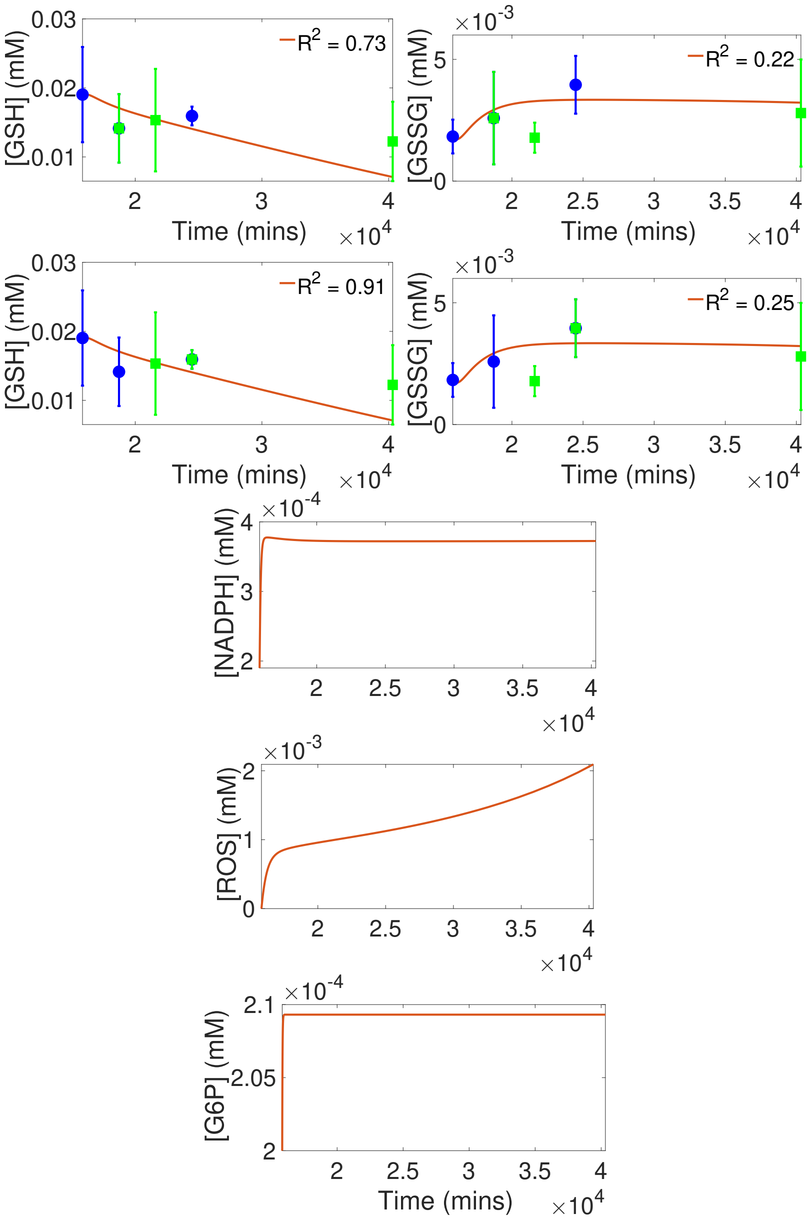

Figure S3: Model calibration and validation for control - case B. $V_{\max_{oxid}} = 1.5043 \times 10^{-1}$, $n_{[\mathrm{GSSG}]} = 1.7202 \times 10^{-4}$. Calibration data shown with circles. Validation data shown with squares.

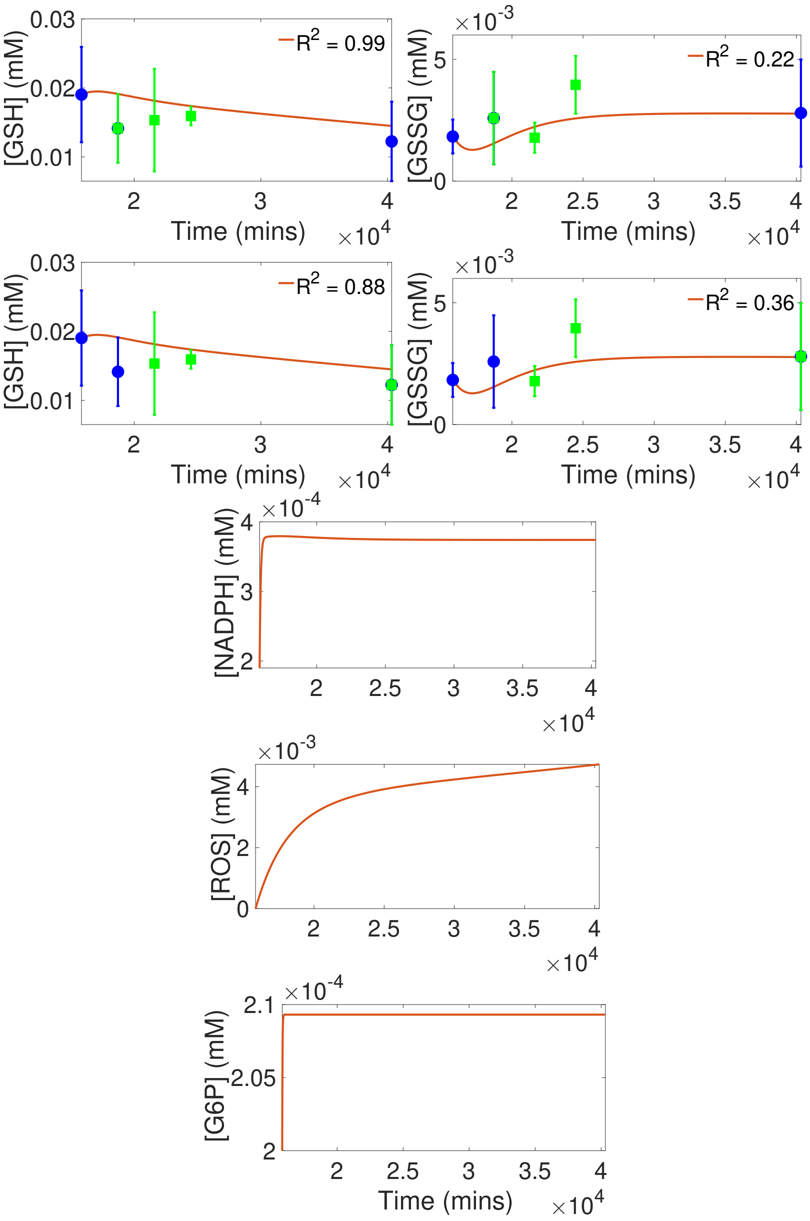

Figure S4: Model calibration and validation for control - case C. $V_{\max_{oxid}} = 2.4566 \times 10^{-2}$, $n_{[\text{GSSG}]} = 8.5432 \times 10^{-5}$. Calibration data shown with circles. Validation data shown with squares.

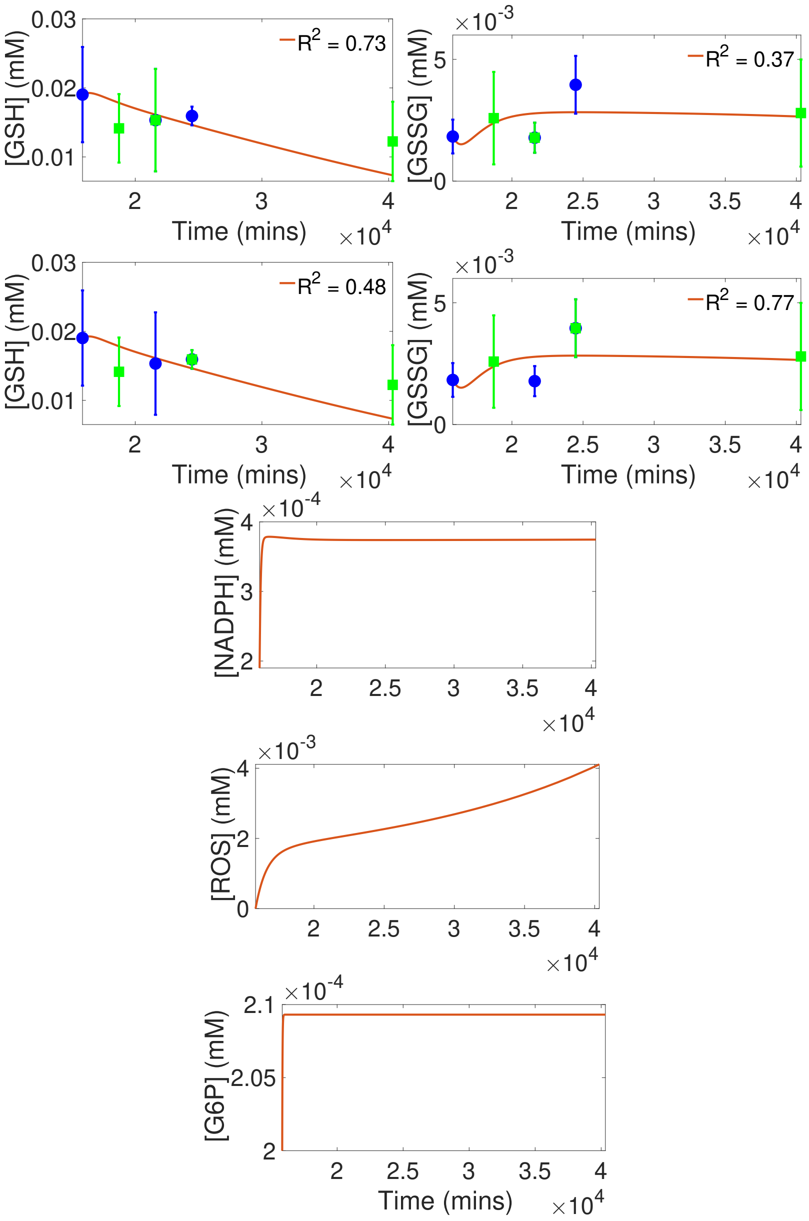

Figure S5: Model calibration and validation for control - case D. $V_{\max_{oxid}} = 7.3370 \times 10^{-2}$, $n_{[\mathrm{GSSG}]} = 3.0335 \times 10^{-4}$. Calibration data shown with circles. Validation data shown with squares.

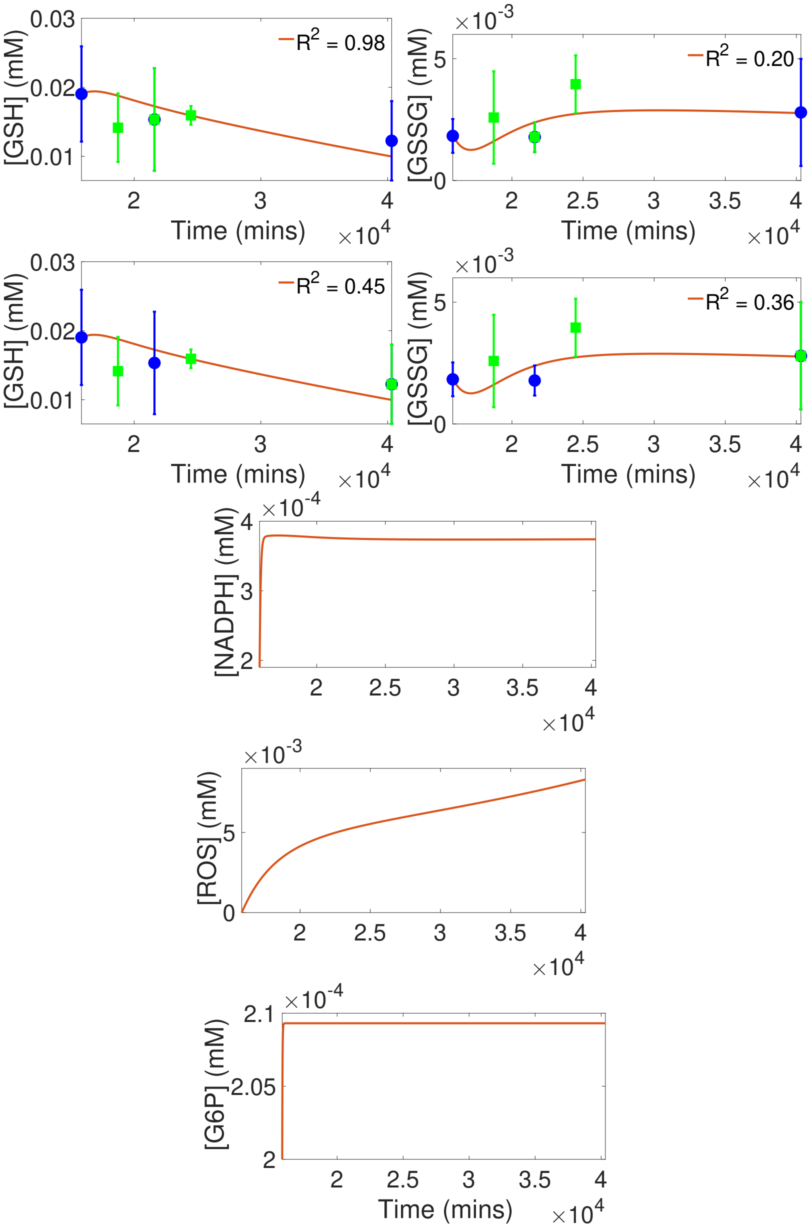

Figure S6: Model calibration and validation for control - case E. $V_{\max_{oxid}} = 2.2679 \times 10^{-2}$, $n_{[\text{GSSG}]} = 1.5735 \times 10^{-4}$. Calibration data shown with circles. Validation data shown with squares.

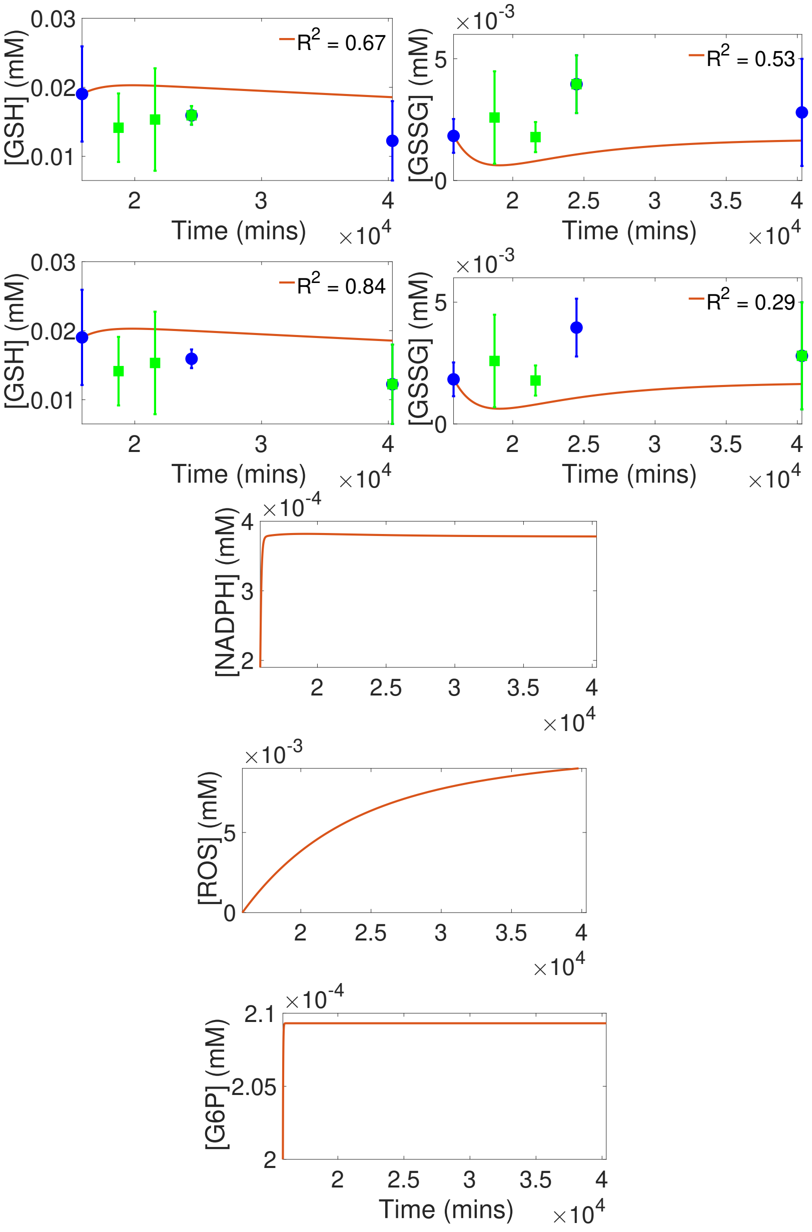

Figure S7: Model calibration and validation for control - case F. $V_{\max_{oxid}} = 6.6193 \times 10^{-3}$, $n_{[\mathrm{GSSG}]} = 1.3662 \times 10^{-4}$. Calibration data shown with circles. Validation data shown with squares.

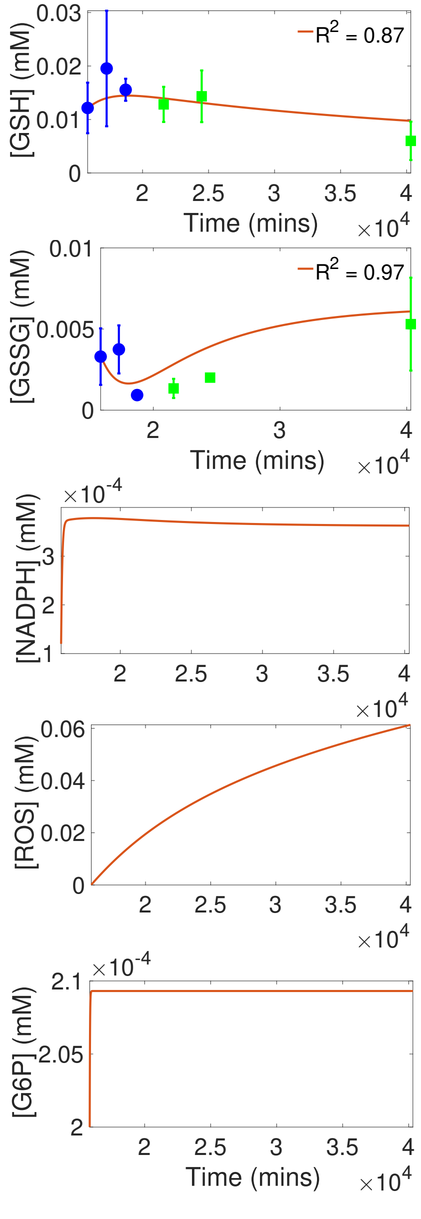

Figure S8: Model calibration and validation for *rd1* - case A. $V_{\max_{oxid}} = 7.5956 \times 10^{-3}$, $n_{[\mathrm{GSSG}]} = 1.2526 \times 10^{-4}$. Calibration data shown with circles. Validation data shown with squares.

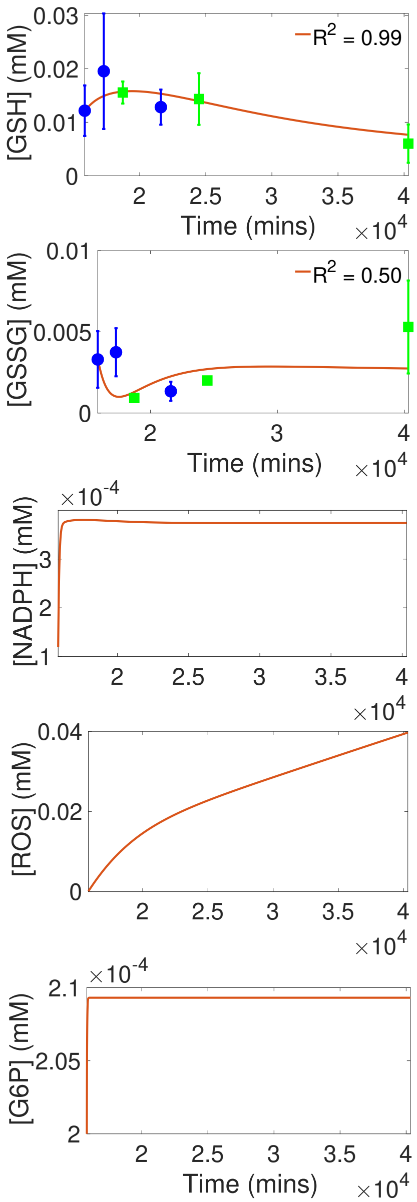

Figure S9: Model calibration and validation for *rd1* - case B. $V_{\max_{oxid}} = 1.4223 \times 10^{-2}$, $n_{[\mathrm{GSSG}]} = 7.8542 \times 10^{-4}$. Calibration data shown with circles. Validation data shown with squares.

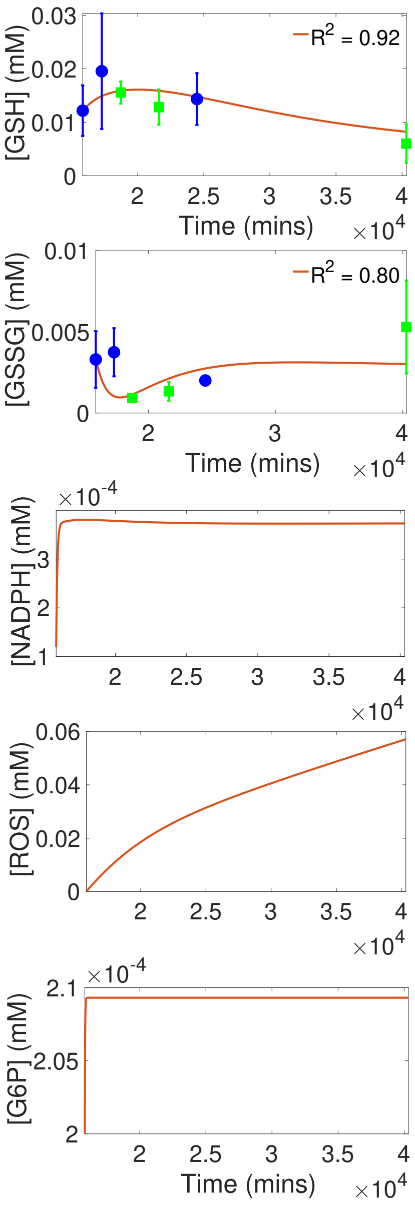

Figure S10: Model calibration and validation for *rd1* - case C. $V_{\max_{oxid}} = 9.0708 \times 10^{-3}$, $n_{[\text{GSSG}]} = 6.5016 \times 10^{-4}$. Calibration data shown with circles. Validation data shown with squares.

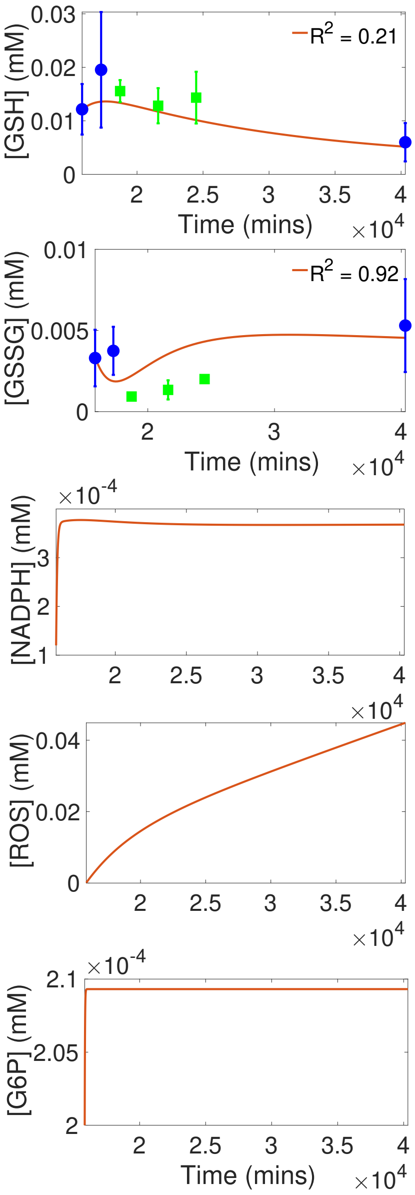

Figure S11: Model calibration and validation for *rd1* - case D. $V_{\max_{oxid}} = 1.6846 \times 10^{-2}$, $n_{[\mathrm{GSSG}]} = 2.2386 \times 10^{-4}$. Calibration data shown with circles. Validation data shown with squares.

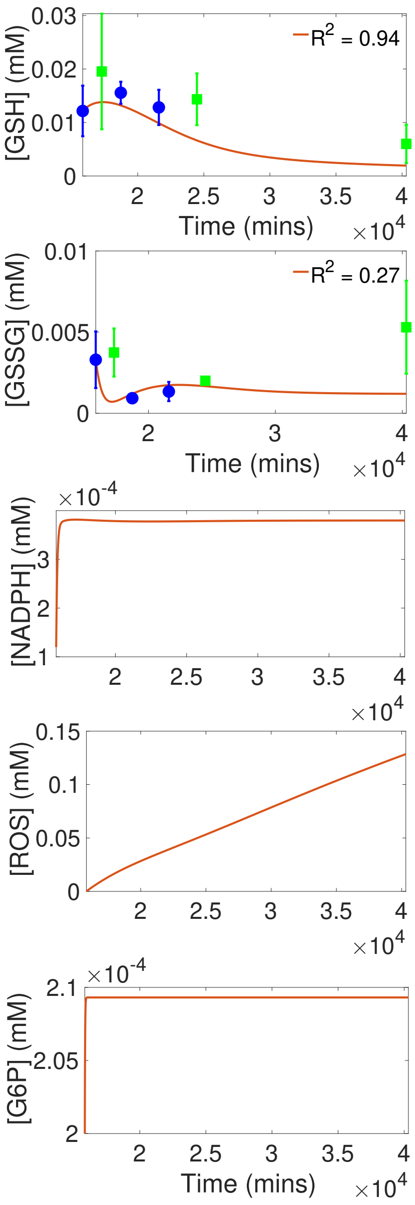

Figure S12: Model calibration and validation for *rd1* - case E. $V_{\max_{oxid}} = 1.2629 \times 10^{-2}$, $n_{[\mathrm{GSSG}]} = 1.5861 \times 10^{-3}$. Calibration data shown with circles. Validation data shown with squares.

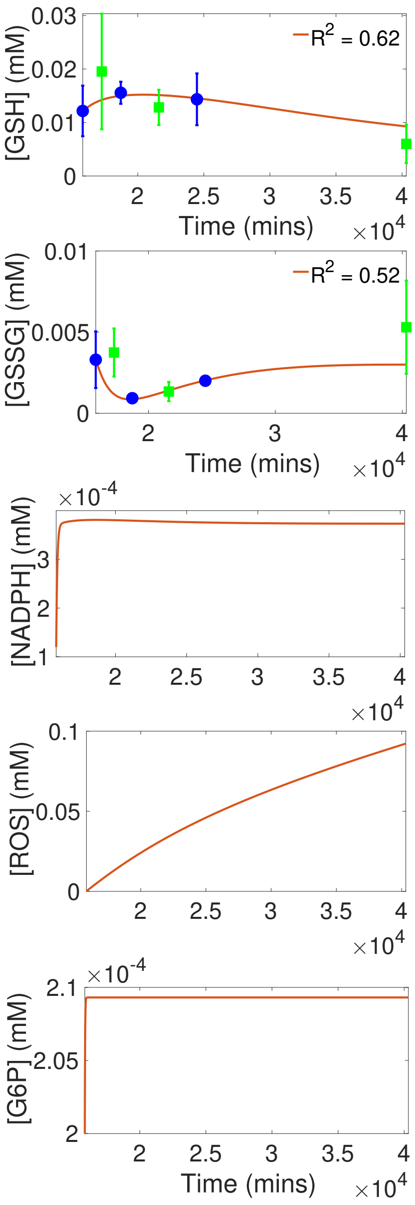

Figure S13: Model calibration and validation for *rd1* - case F. $V_{\max_{oxid}} = 3.7822 \times 10^{-3}$, $n_{[\mathrm{GSSG}]} = 3.7863 \times 10^{-4}$. Calibration data shown with circles. Validation data shown with squares.

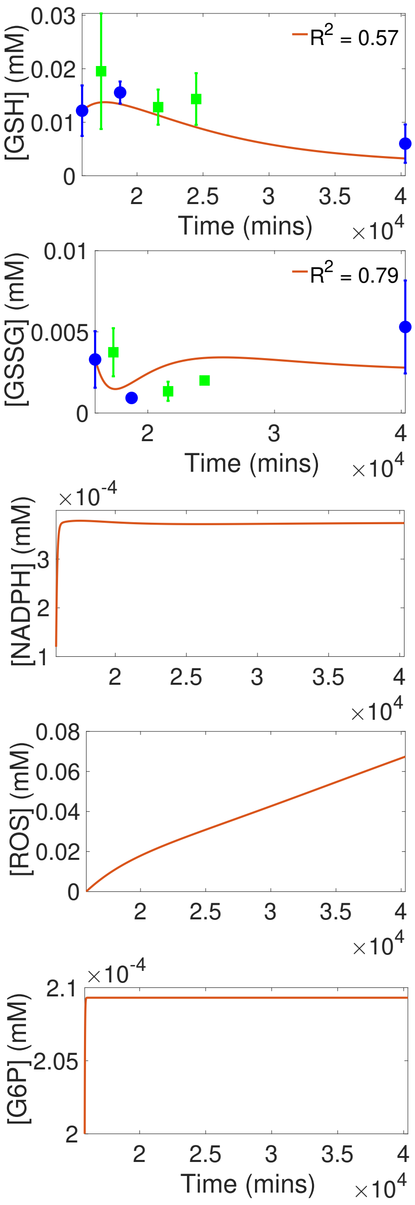

Figure S14: Model calibration and validation for *rd1* - case G. $V_{\max_{oxid}} = 1.5315 \times 10^{-2}$, $n_{[\mathrm{GSSG}]} = 4.8178 \times 10^{-4}$. Calibration data shown with circles. Validation data shown with squares.

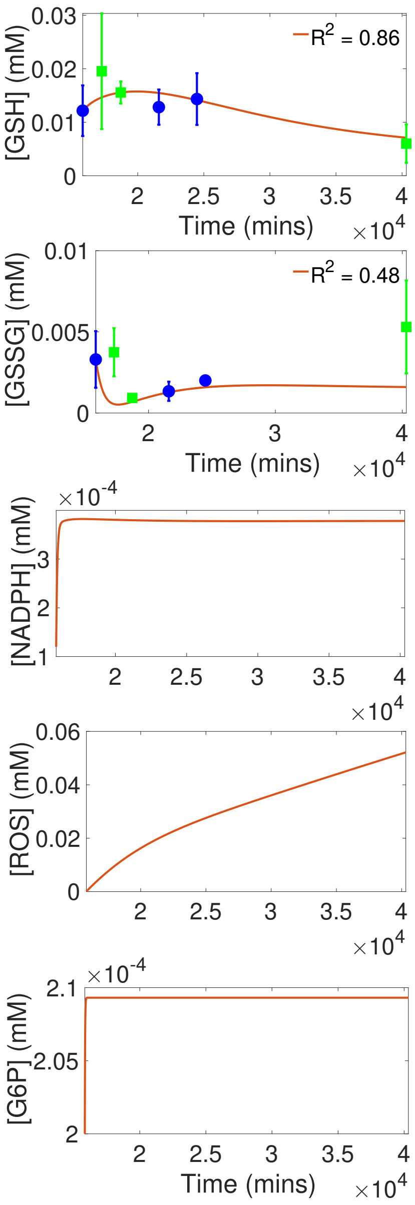

Figure S15: Model calibration and validation for *rd1* - case H. $V_{\max_{oxid}} = 9.3293 \times 10^{-3}$, $n_{[\mathrm{GSSG}]} = 1.2433 \times 10^{-3}$. Calibration data shown with circles. Validation data shown with squares.

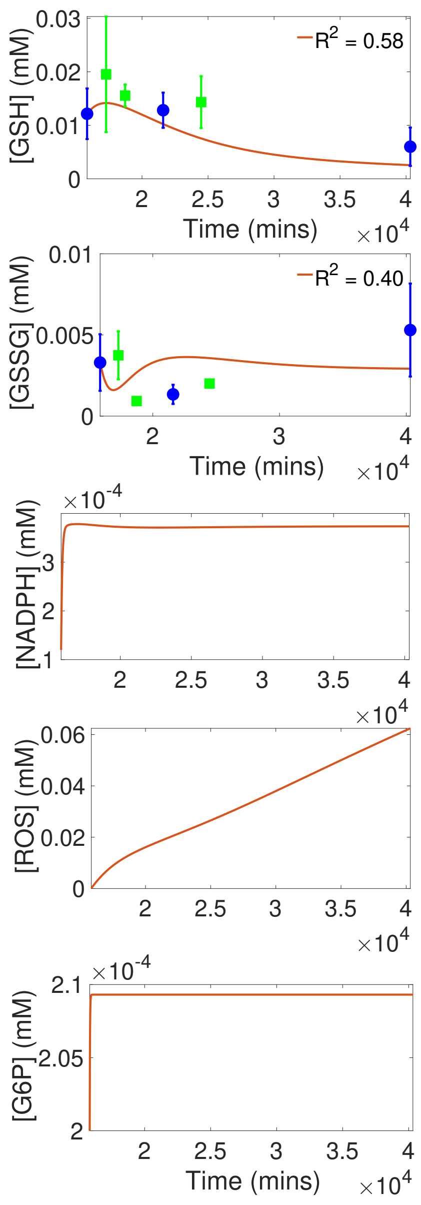

Figure S16: Model calibration and validation for *rd1* - case I. $V_{\text{max}_{oxid}} = 3.0570 \times 10^{-2}$, $n_{[\text{GSSG}]} = 8.5949 \times 10^{-4}$. Calibration data shown with circles. Validation data shown with squares.

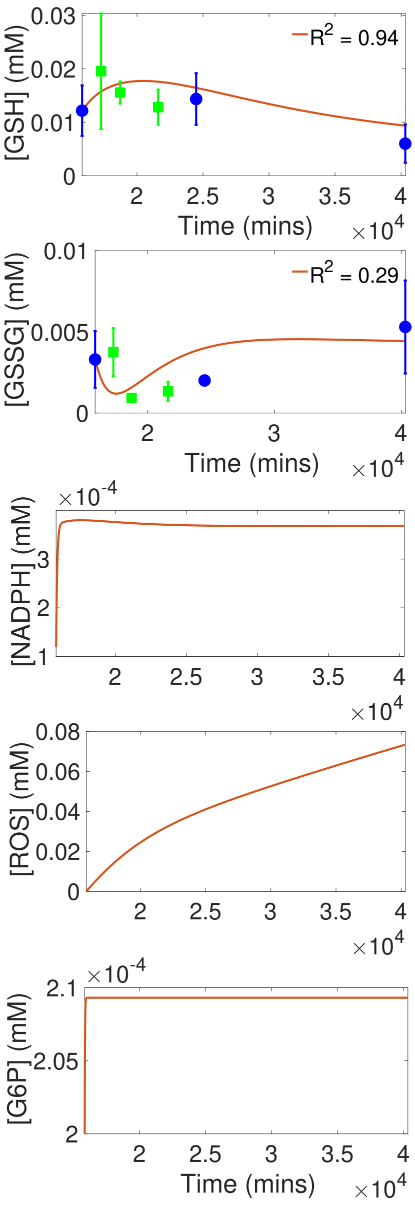

Figure S17: Model calibration and validation for *rd1* - case J. $V_{\max_{oxid}} = 8.6242 \times 10^{-3}$, $n_{[\mathrm{GSSG}]} = 5.9505 \times 10^{-4}$. Calibration data shown with circles. Validation data shown with squares.

Table S3: PRCC values for normal and RP conditions (given in parentheses for each input factor; included are only the input factors to which the outcomes of interest are sensitive to). PN: postnatal day. Note that the parameters listed in this table are the same as those in Table 4 in the manuscript, but this table also includes the PRCC value for each parameter.

Control conditions

(A) Outcome of interest [GSH]/[GSSG]

PN	PRCC values
12	$r(-0.77)$, $[\mathrm{GSH}]_0(0.75)$, $V\mathrm{max}_{oxid}(-0.70)$, $\theta_{[\mathrm{ROS}]}(0.53)$, $Ks_{[\mathrm{GSSG}]}(-0.51)$
13	$r(-0.81)$, $\theta_{[\mathrm{ROS}]}(0.63)$, $[\mathrm{GSH}]_0(0.60)$, $V_p(0.59)$, $Ks_{[\mathrm{GSSG}]}(-0.55)$
15	$r(-0.82)$, $\theta_{[\mathrm{ROS}]}(0.63)$, $V_p(0.61)$, $n_{[\mathrm{NADPH}]}(-0.60)$, $[\mathrm{GSH}]_0(0.597)$, $Ks_{[\mathrm{GSSG}]}(-0.53)$
17	$r(-0.82)$, $\theta_{[\mathrm{ROS}]}(0.68)$, $[\mathrm{GSH}]_0(0.583)$, $V_p(0.582)$, $n_{[\mathrm{NADPH}]}(-0.53)$
28	$r(-0.80)$, $\theta_{[\mathrm{ROS}]}(0.63)$, $Ks_{[\mathrm{GSSG}]}(-0.57)$, $n_{[\mathrm{NADPH}]}(-0.55)$, $V_p(0.51)$

(B) Outcome of interest [NADPH]

PN	PRCC values
12	$V_p(0.96)$, $n_{[\mathrm{NADPH}]}(-0.95)$
13	$V_p(0.94)$, $n_{[\mathrm{NADPH}]}(-0.93)$
15	$V_p(0.95)$, $n_{[\mathrm{NADPH}]}(-0.94)$
17	$V_p(0.96)$, $n_{[\mathrm{NADPH}]}(-0.95)$
28	$V_p(0.95)$, $n_{[\mathrm{NADPH}]}(-0.94)$

(C) Outcome of interest [ROS]

PN	PRCC values
12	$V\mathrm{max}_{oxid}(-0.85)$, $r(0.83)$, $[\mathrm{GSH}]_0(-0.75)$, $Km_{[\mathrm{GSH}]}(0.70)$, $\theta_{[\mathrm{ROS}]}(-0.68)$, $Ks_{[\mathrm{ROS}]}(0.679)$
13	$V\mathrm{max}_{oxid}(-0.85)$, $r(0.82)$, $[\mathrm{GSH}]_0(-0.69)$, $Ks_{[\mathrm{ROS}]}(0.66)$, $Km_{[\mathrm{GSH}]}(0.65)$, $\theta_{[\mathrm{ROS}]}(-0.64)$
15	$r(0.83)$, $V\mathrm{max}_{oxid}(-0.80)$, $\theta_{[\mathrm{ROS}]}(-0.72)$, $Km_{[\mathrm{GSH}]}(0.61)$, $[\mathrm{GSH}]_0(-0.60)$, $Ks_{[\mathrm{ROS}]}(0.56)$
17	$r(0.82)$, $V\mathrm{max}_{oxid}(-0.74)$, $\theta_{[\mathrm{ROS}]}(-0.67)$, $[\mathrm{GSH}]_0(-0.58)$
28	$r(0.83)$, $\theta_{[\mathrm{ROS}]}(-0.67)$, $s_{[\mathrm{GSH}]}(-0.57)$, $V\mathrm{max}_{oxid}(-0.53)$

(D) Outcome of interest [G6P]

PN	PRCC values
12	$K_n(0.95)$, $V_p(0.84)$, $\Phi(-0.83)$, $V_n(-0.78)$
13	$K_n(0.96)$, $\Phi(-0.85)$, $V_p(0.846)$, $V_n(-0.82)$
15	$K_n(0.96)$, $V_p(0.89)$, $V_n(-0.88)$, $\Phi(-0.87)$
17	$K_n(0.94)$, $V_n(-0.80)$, $V_p(0.78)$, $\Phi(-0.77)$
28	$K_n(0.95)$, $V_p(0.84)$, $\Phi(-0.822)$, $V_n(-0.821)$

RP conditions

(A) Outcome of interest [GSH]/[GSSG]

PN	PRCC values
12	$n_{[\mathrm{GSSG}]}(0.74)$, $V\mathrm{max}_{oxid}(-0.62)$, $r(-0.56)$, $Ks_{[\mathrm{ROS}]}(0.559)$, $Km_{[\mathrm{GSH}]}(0.552)$
13	$V\mathrm{max}_{oxid}(-0.85)$, $r(-0.75)$, $Km_{[\mathrm{GSH}]}(0.74)$, $n_{[\mathrm{GSSG}]}(0.69)$, $Ks_{[\mathrm{ROS}]}(0.68)$
15	$V\mathrm{max}_{oxid}(-0.82)$, $r(-0.74)$, $Km_{[\mathrm{GSH}]}(0.63)$, $n_{[\mathrm{GSSG}]}(0.58)$, $Ks_{[\mathrm{ROS}]}(0.56)$, $n_{[\mathrm{NADPH}]}(-0.5004)$
17	$V\mathrm{max}_{oxid}(-0.86)$, $r(-0.75)$, $Km_{[\mathrm{GSH}]}(0.66)$, $Ks_{[\mathrm{ROS}]}(0.62)$, $n_{[\mathrm{NADPH}]}(-0.54)$, $n_{[\mathrm{GSSG}]}(0.5398)$
28	$r(-0.82)$, $V\mathrm{max}_{oxid}(-0.79)$, $Ks_{[\mathrm{ROS}]}(0.68)$, $Km_{[\mathrm{GSH}]}(0.64)$, $Ks_{[\mathrm{GSSG}]}(-0.56)$, $n_{[\mathrm{NADPH}]}(-0.53)$, $V_p(0.5005)$

(B) Outcome of interest [NADPH]

PN	PRCC values
12	$V_p(0.9444)$, $n_{[\mathrm{NADPH}]}(-0.9443)$
13	$V_p(0.955)$, $n_{[\mathrm{NADPH}]}(-0.952)$
15	$V_p(0.958)$, $n_{[\mathrm{NADPH}]}(-0.956)$
17	$V_p(0.95)$, $n_{[\mathrm{NADPH}]}(-0.94)$
28	$V_p(0.9423)$, $n_{[\mathrm{NADPH}]}(-0.9418)$

(C) Outcome of interest [ROS]

PN	PRCC values
12	$r(0.99)$, $V\mathrm{max}_{oxid}(-0.61)$, $Ks_{[\mathrm{ROS}]}(0.58)$, $Km_{[\mathrm{GSH}]}(0.53)$
13	$r(0.99)$, $V\mathrm{max}_{oxid}(-0.73)$, $Km_{[\mathrm{GSH}]}(0.67)$, $Ks_{[\mathrm{ROS}]}(0.62)$, $[\mathrm{GSH}]_0(-0.60)$, $\theta_{[\mathrm{ROS}]}(-0.504)$
15	$r(0.98)$, $V\mathrm{max}_{oxid}(-0.72)$, $\theta_{[\mathrm{ROS}]}(-0.65)$, $Ks_{[\mathrm{ROS}]}(0.56)$, $[\mathrm{GSH}]_0(-0.54)$, $Km_{[\mathrm{GSH}]}(0.52)$
17	$r(0.97)$, $V\mathrm{max}_{oxid}(-0.64)$, $\theta_{[\mathrm{ROS}]}(-0.58)$, $[\mathrm{GSH}]_0(-0.51)$
28	$r(0.97)$, $\theta_{[\mathrm{ROS}]}(-0.60)$, $s_{[\mathrm{GSH}]}(-0.595)$, $n_{[\mathrm{GSSG}]}(0.56)$

(D) Outcome of interest [G6P]

PN	PRCC values
12	$K_n(0.96)$, $V_p(0.87)$, $\Phi(-0.86)$, $V_n(-0.857)$
13	$K_n(0.94)$, $V_p(0.84)$, $\Phi(-0.82)$, $V_n(-0.77)$
15	$K_n(0.96)$, $V_p(0.845)$, $V_n(-0.841)$, $\Phi(-0.83)$
17	$K_n(0.95)$, $\Phi(-0.84)$, $V_n(-0.837)$, $V_p(0.79)$
28	$K_n(0.95)$, $V_n(-0.84)$, $\Phi(-0.813)$, $V_p(0.8103)$
